# Supplementary material for: Discovery of Fungus-Specific Targets and Inhibitors Using Chemical Phenotyping of Pathogenic Spore Germination
Source: mBio. 2021 Jul 27;12(4):e01672-21. doi: 10.1128/mBio.01672-21 (PMC8406298; doi:10.1128/mBio.01672-21)
Supplement: FIG S1 [file mbio.01672-21-sf001.pdf]

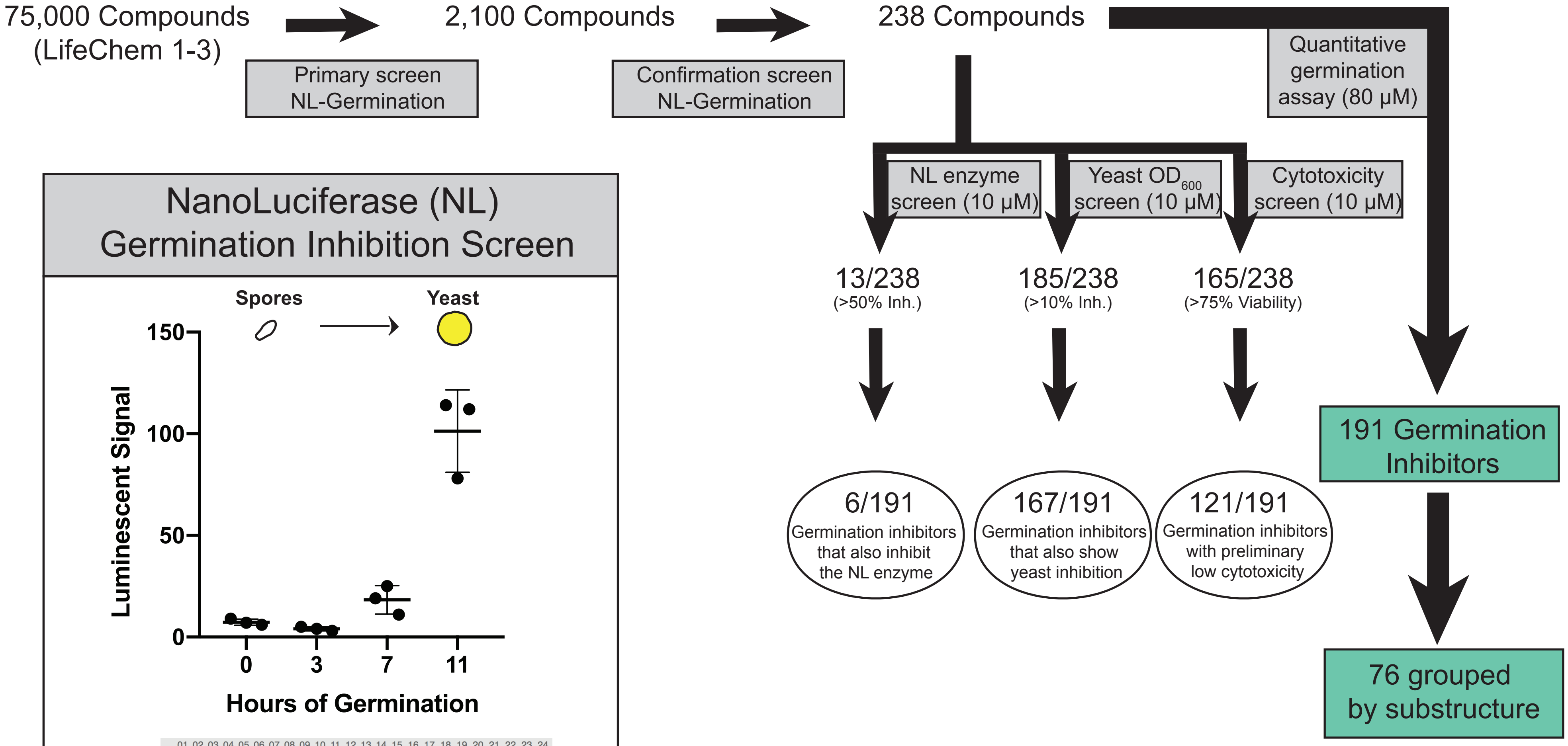

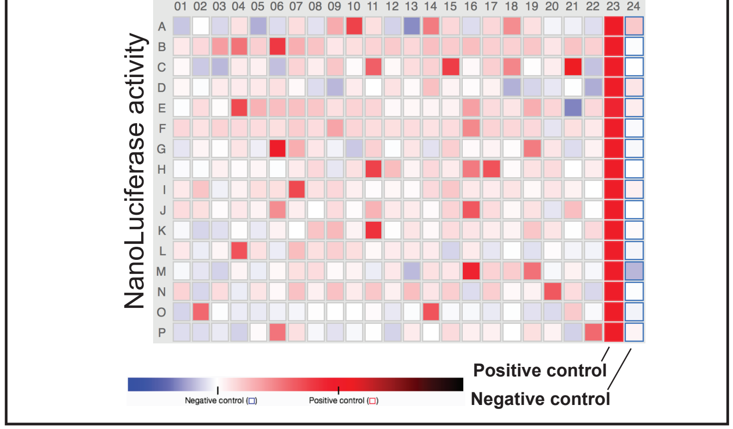

Figure S1. Description of assay and flow chart of high throughput screening process for germination inhibitors. Box (top): spores containing NanoLuciferase (NL) fusion construct demonstrate an increase in NL signal over the course of germination. X-axis represents hours of germination and Y-axis represents luminescent signal (error bars represent standard deviation among 3 independent replicates). Box (bottom): representation of a 384-well screening plate containing drug-like compounds. Red indicates germination inhibition. Positive control for inhibition (column 23) contained 3.55 μM cycloheximide. Negative control (column 24) contained 0.1% DMSO (carrier only).
