# Supplementary material for: Discovery of Fungus-Specific Targets and Inhibitors Using Chemical Phenotyping of Pathogenic Spore Germination
Source: mBio. 2021 Jul 27;12(4):e01672-21. doi: 10.1128/mBio.01672-21 (PMC8406298; doi:10.1128/mBio.01672-21)
Supplement: DATA SET S2 [file mbio.01672-21-sd002.pdf]

PubChem CID	Structure	Control 0 Hours	Control 12 Hours	80 µM 0 Hours	80 µM 12 Hours	Germination Inhibitor	Compound Precipitation	Relative Level of Inhibition
17520	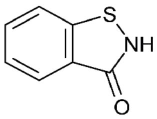	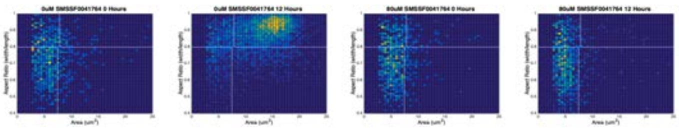				Yes	No	Full
27144	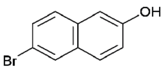	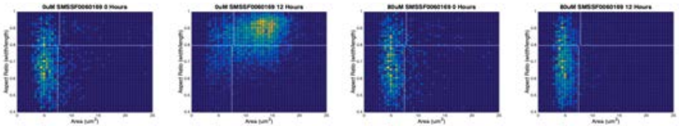				Yes	No	Full
27297	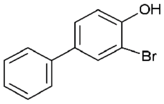	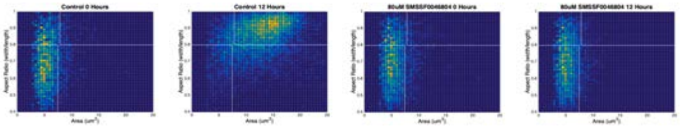				Yes	No	Full
66123	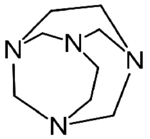	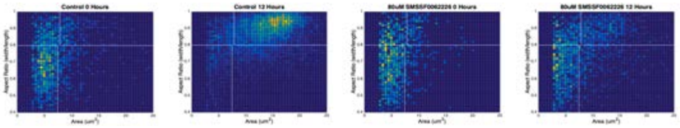				Yes	No	Intermediate
76937	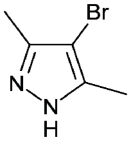	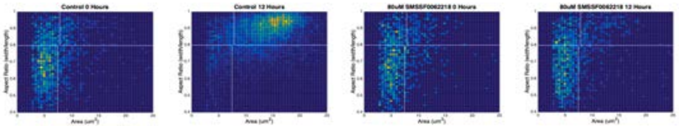				Yes	No	Full
99741	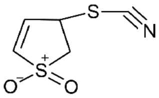	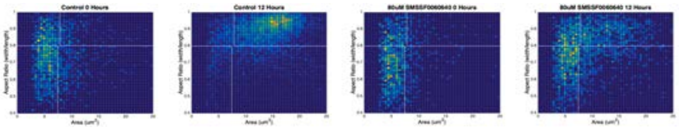				Yes	No	Intermediate
121637	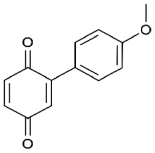	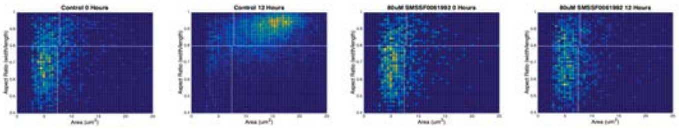				Yes	No	Full
243059	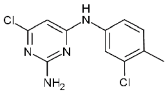	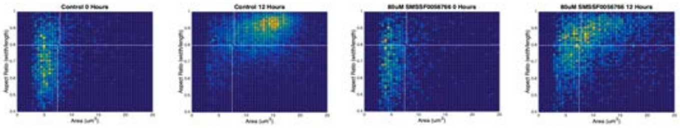				Yes	No	Intermediate
282938	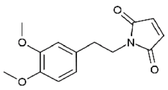	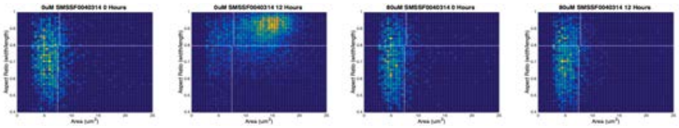				Yes	No	Full
292929	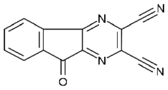	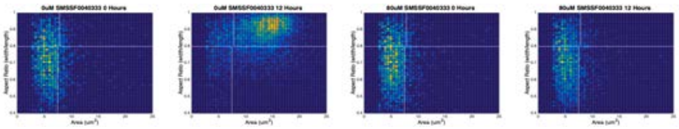				Yes	No	Full

302279	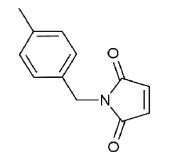	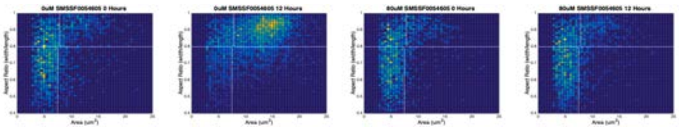	Yes	No	Full
310131	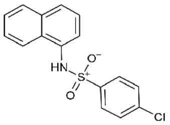	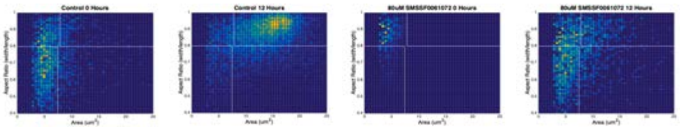	Yes	Yes	Debris
652808	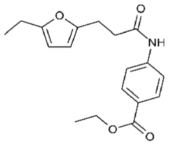	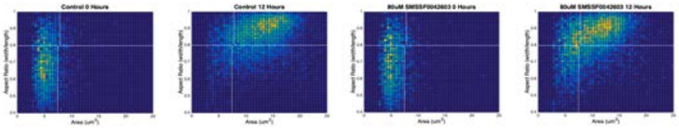	Yes	No	Intermediate
673711	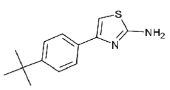	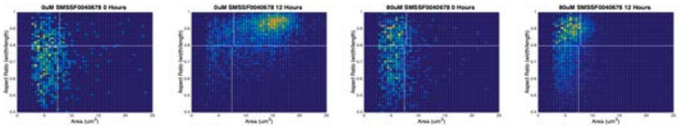	Yes	No	Intermediate
688709	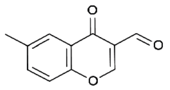	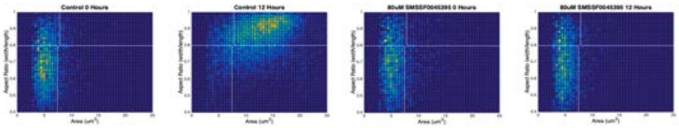	Yes	No	Full
697528	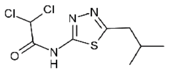	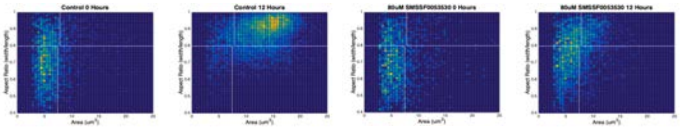	Yes	No	Intermediate
700009	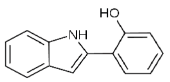	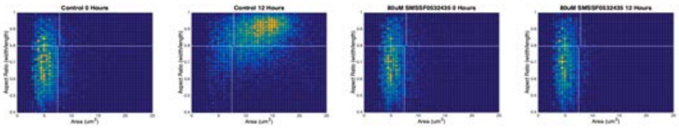	Yes	No	Full
700148	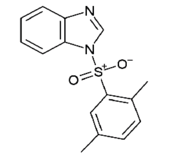	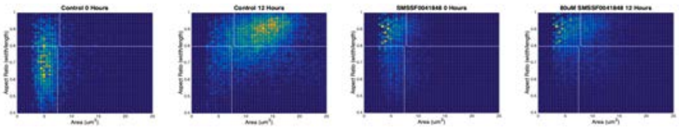	Yes	Yes	Debris
700976	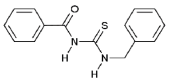	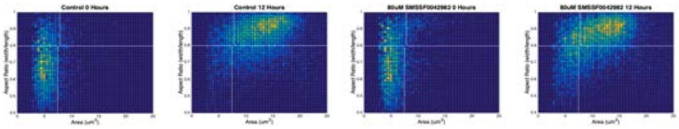	Yes	No	Intermediate
701070	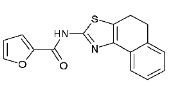	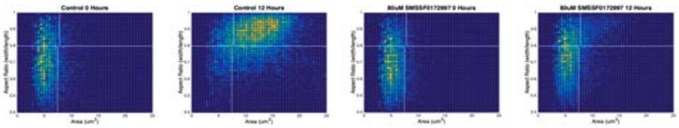	Yes	No	Full
707936	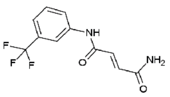	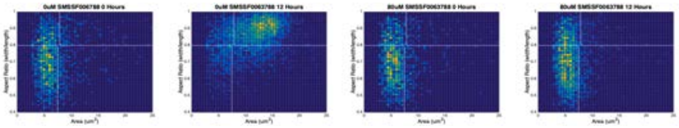	Yes	No	Full

709153	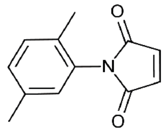	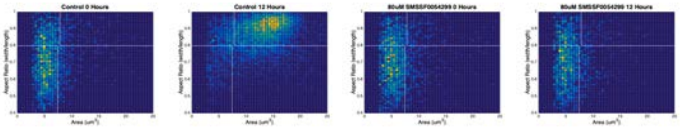	Yes	No	Full
717711	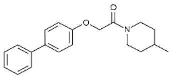	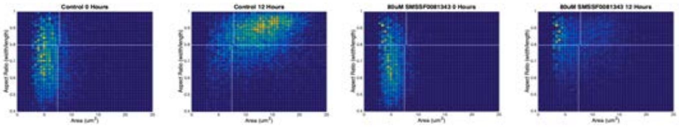	Yes	Yes	Debris
723732	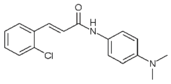	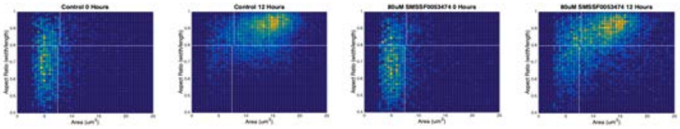	Yes	No	Intermediate
731806	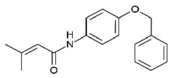	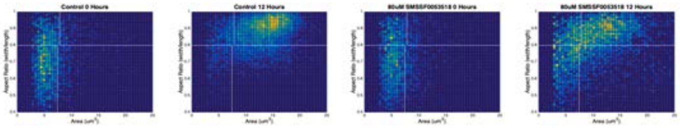	Yes	No	Intermediate
733834	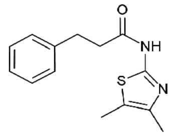	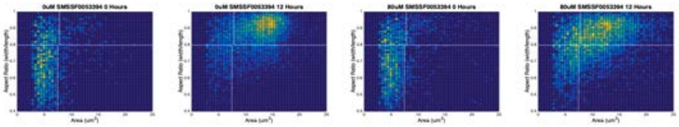	Yes	No	Intermediate
749608	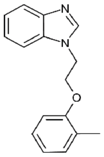	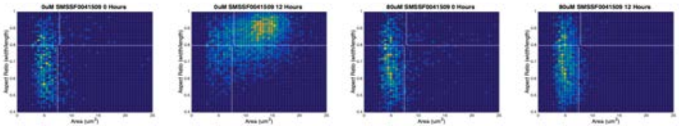	Yes	No	Full
766532	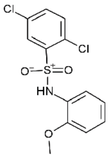	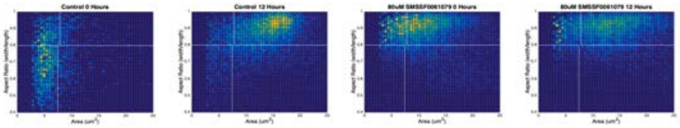	Yes	Yes	Debris
767639	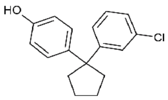	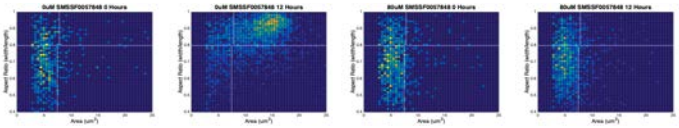	Yes	No	Full
768672	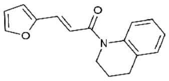	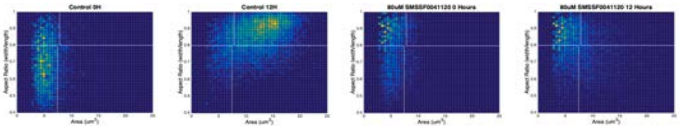	Yes	Yes	Debris
773093	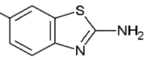	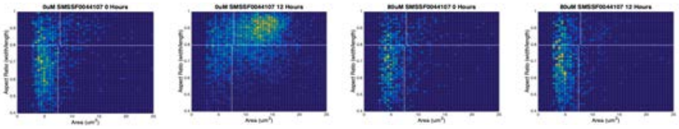	Yes	No	Full
780598	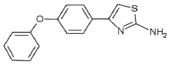	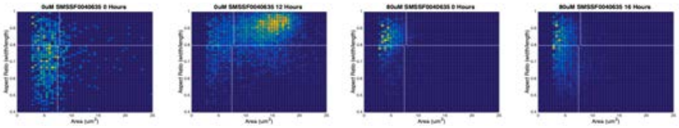	Yes	Yes	Debris

790960	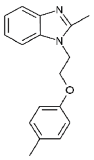	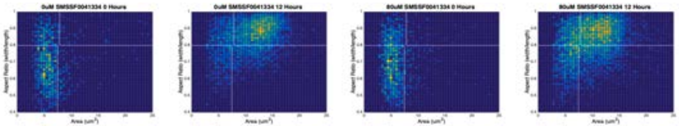	Yes	No	Intermediate
803194	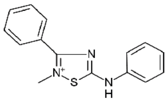	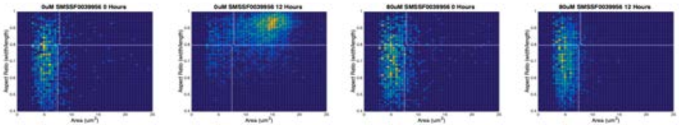	Yes	No	Full
820752	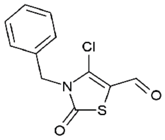	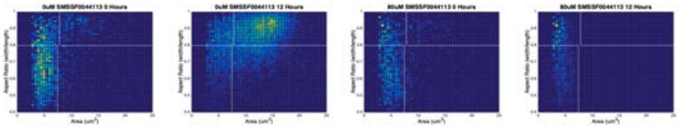	Yes	Yes	Debris
852322	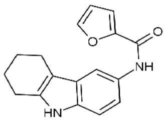	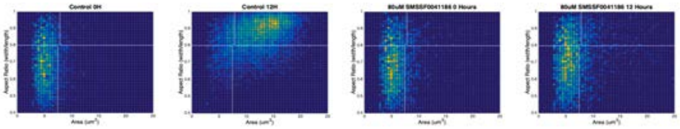	Yes	No	Full
874714	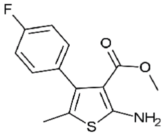	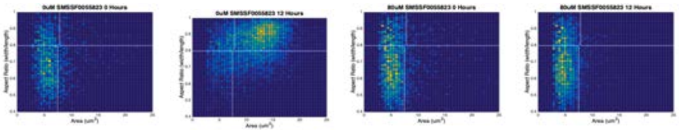	Yes	No	Full
889748	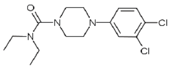	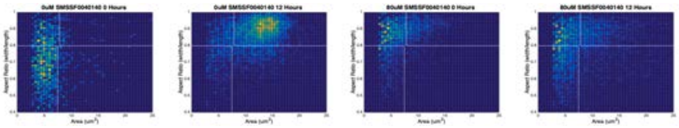	Yes	Yes	Debris
899259	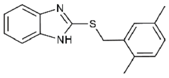	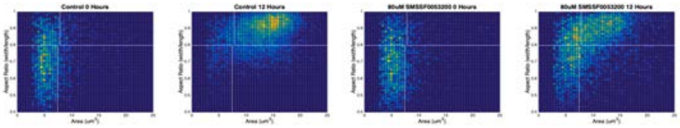	Yes	No	Intermediate
944383	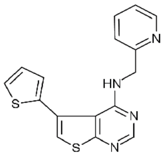	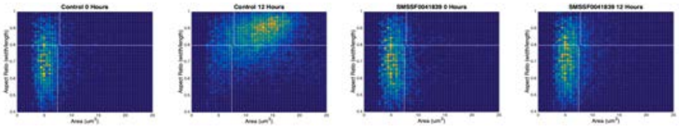	Yes	No	Full
955598	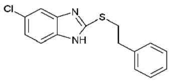	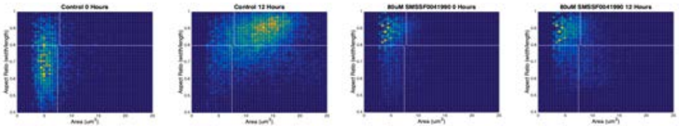	Yes	Yes	Debris
957599	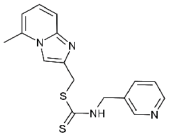	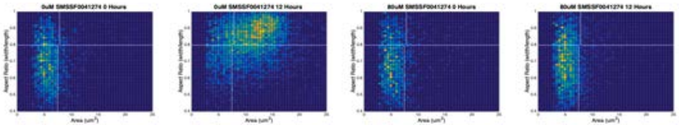	Yes	No	Full
962333	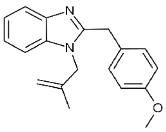	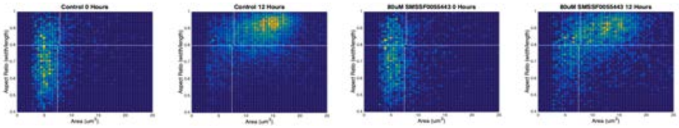	Yes	No	Intermediate

973573	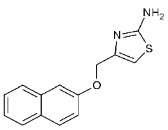	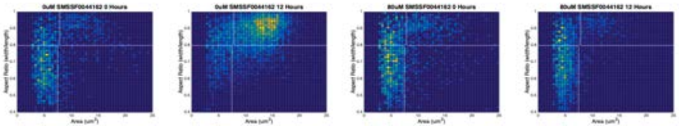	Yes	No	Full
983861	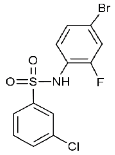	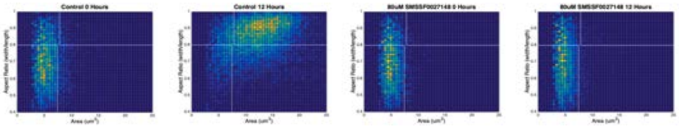	Yes	No	Full
1092459	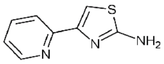	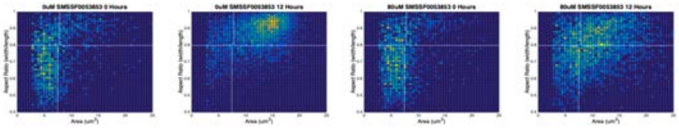	Yes	No	Intermediate
1186249	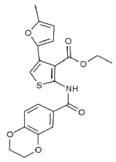	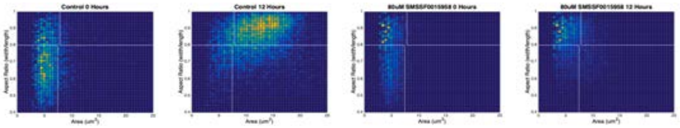	Yes	Yes	Debris
1204290	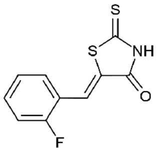	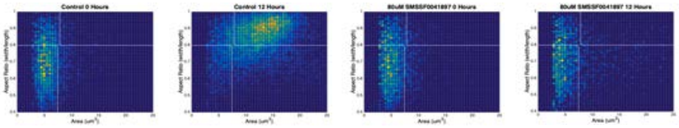	Yes	No	Full
1307471	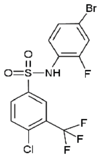	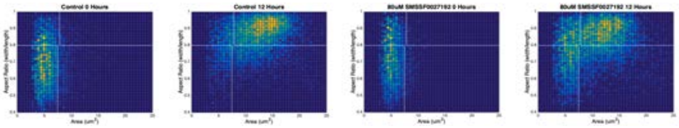	Yes	No	Intermediate
1391595	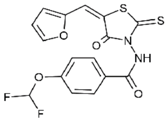	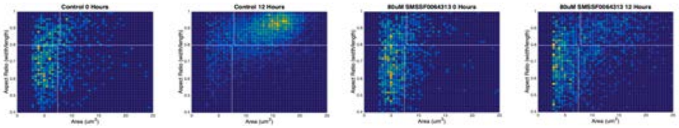	Yes	no	Intermediate
1475928	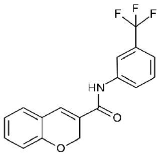	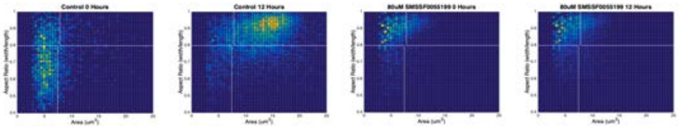	Yes	Yes	Debris
1505880	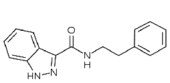	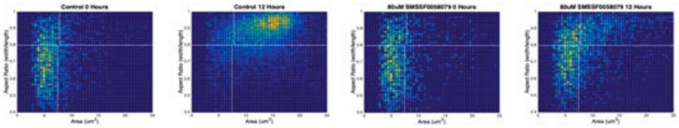	Yes	No	Intermediate
1570496	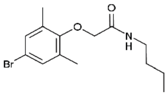	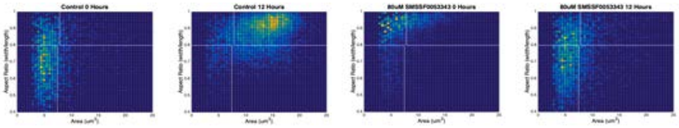	Yes	Yes	Debris
1589778	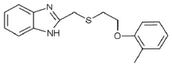	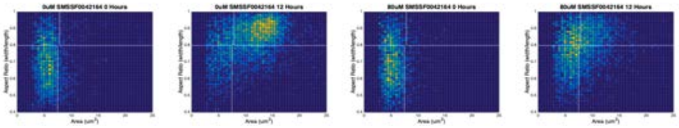	Yes	No	Intermediate

1589789	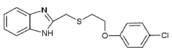	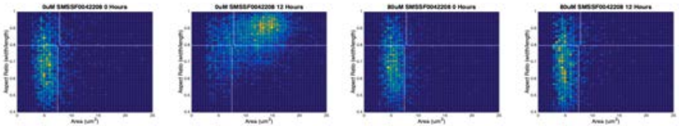	Yes	No	Full
1636480	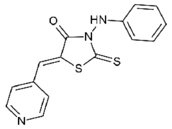	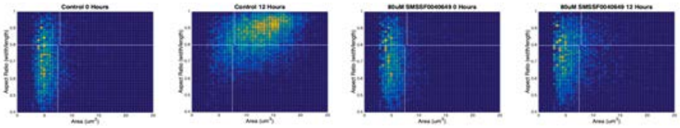	Yes	No	Full
1653621	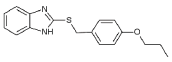	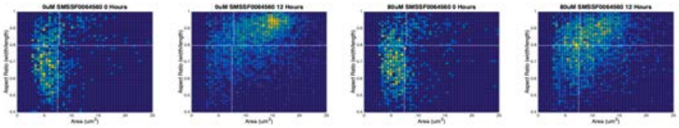	Yes	No	Intermediate
1653635	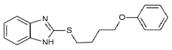	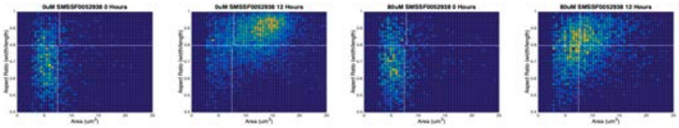	Yes	No	Intermediate
1992009	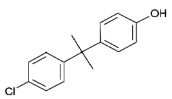	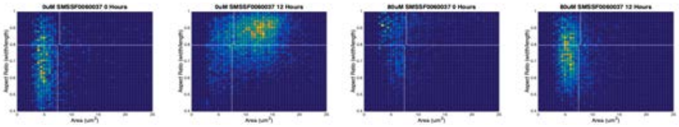	Yes	Yes	Debris
2008712	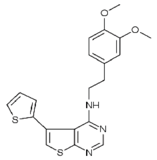	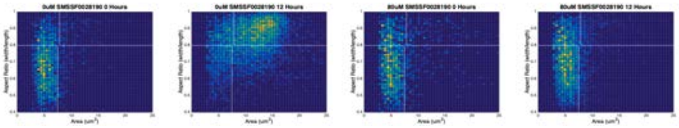	Yes	No	Full
2049334	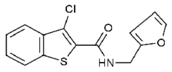	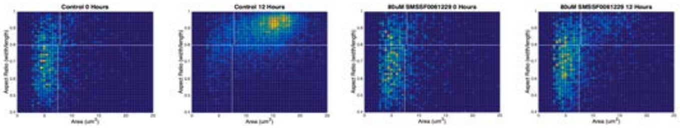	Yes	No	Full
2049855	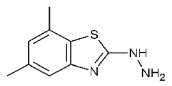	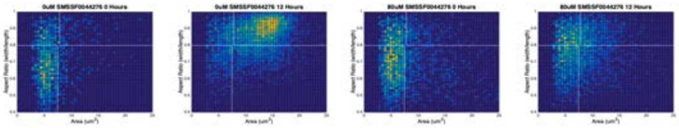	Yes	No	Intermediate
2129690	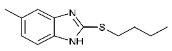	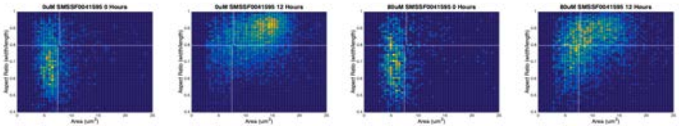	Yes	No	Intermediate
2133779	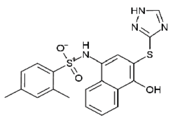	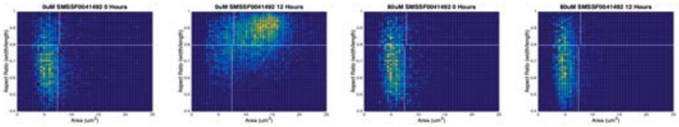	Yes	No	Full
2139195	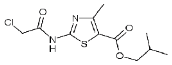	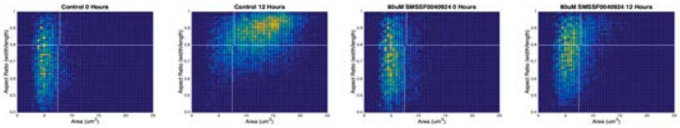	Yes	No	Full

2238990	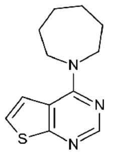	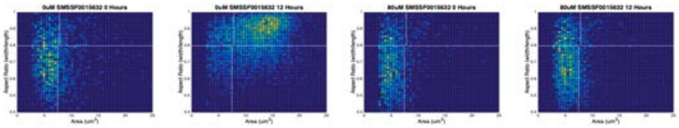	Yes	No	Full
2238992	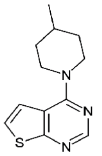	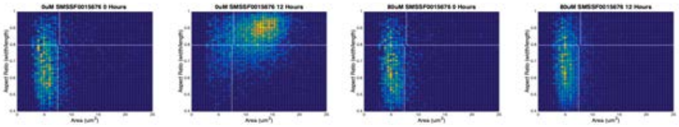	Yes	No	Full
2238994	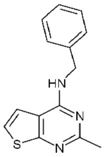	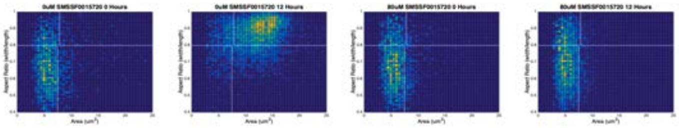	Yes	No	Full
2239015	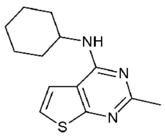	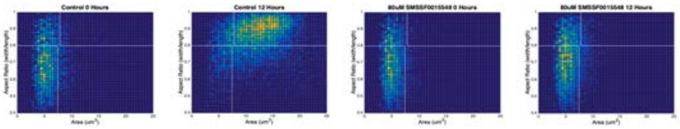	Yes	No	Full
2354424	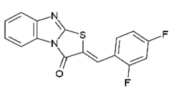	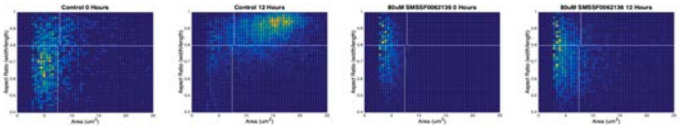	Yes	Yes	Debris
2497352	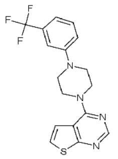	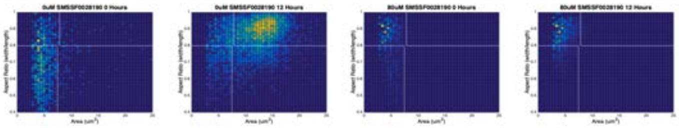	Yes	Yes	Debris
2769740	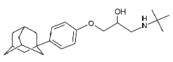	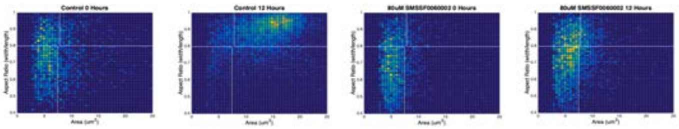	Yes	No	Intermediate
2771030	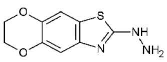	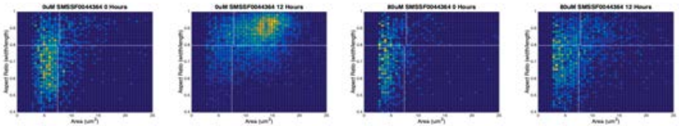	Yes	No	Intermediate
2793380	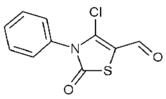	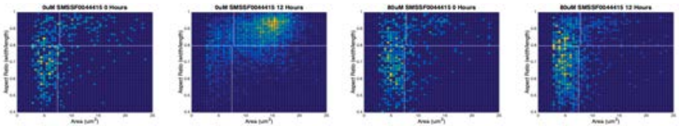	Yes	No	Full
2831154	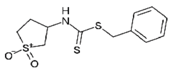	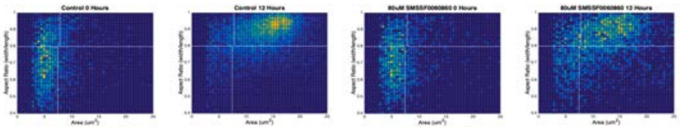	Yes	no	Intermediate
2834581	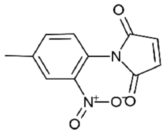	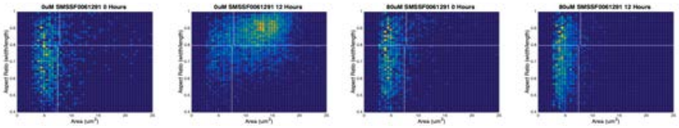	Yes	No	Full

2919713	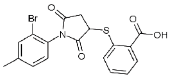	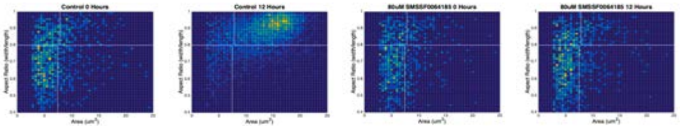	Yes	No	Full
2936975	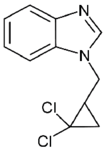	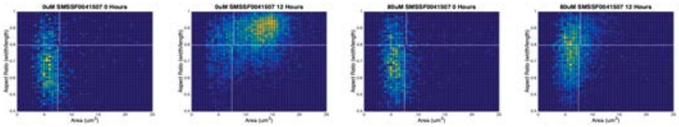	Yes	No	Intermediate
2943193	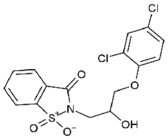	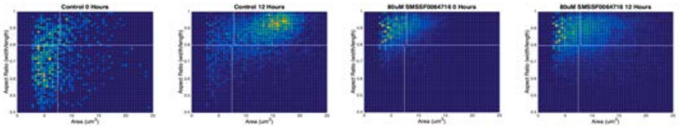	Yes	Yes	Debris
2954695	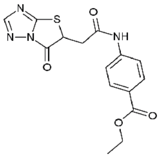	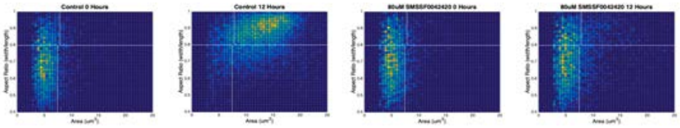	Yes	No	Full
2974037	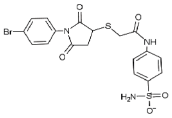	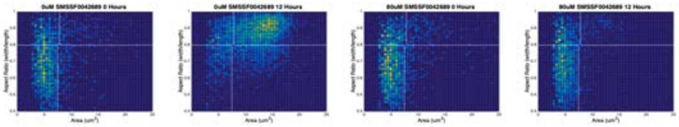	Yes	No	Full
2981975	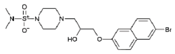	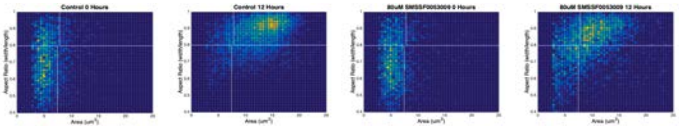	Yes	No	Intermediate
2992099	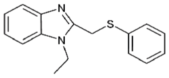	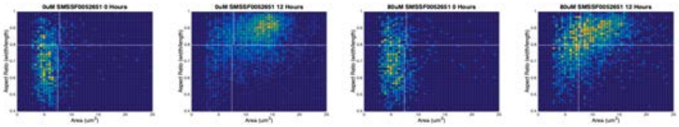	Yes	No	Intermediate
3090522	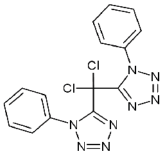	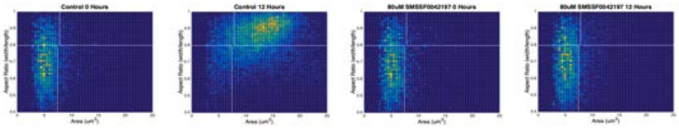	Yes	No	Full
3163780	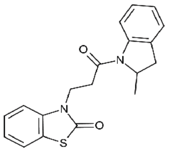	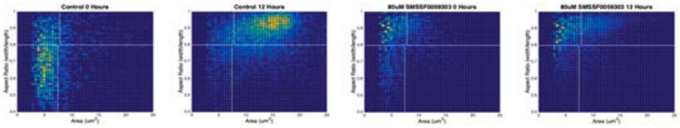	Yes	Yes	Debris
3242906	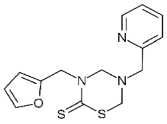	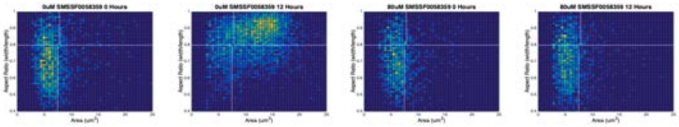	Yes	No	Full
3287596	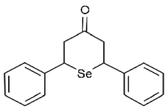	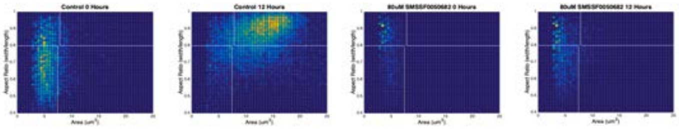	Yes	Yes	Debris

3377203	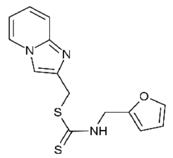	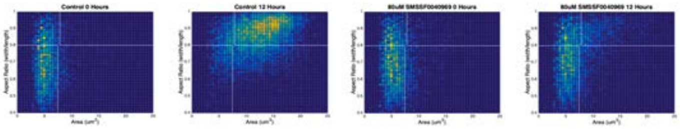	Yes	No	Intermediate
3397555	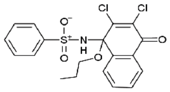	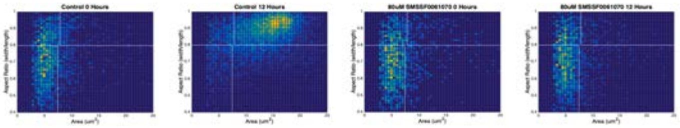	Yes	No	Full
3516311	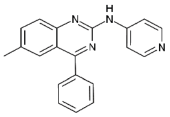	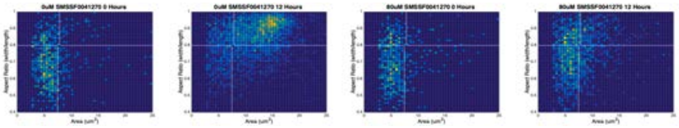	Yes	No	Full
3521356	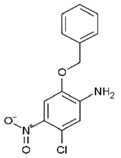	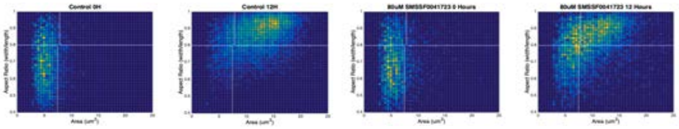	Yes	No	Intermediate
3624343	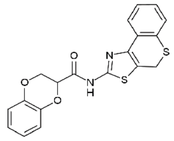	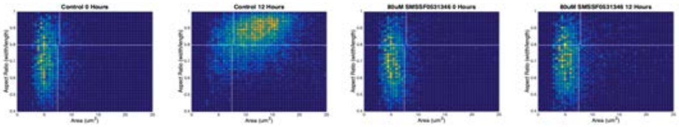	Yes	No	Full
3797566	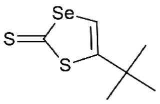	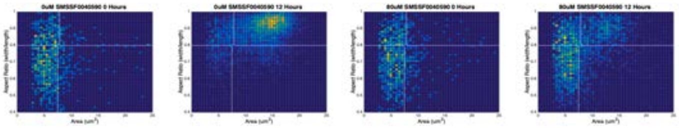	Yes	No	Intermediate
4081132	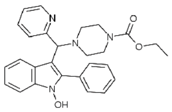	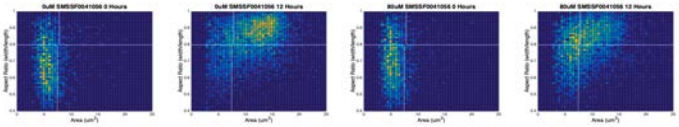	Yes	No	Intermediate
4086498	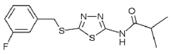	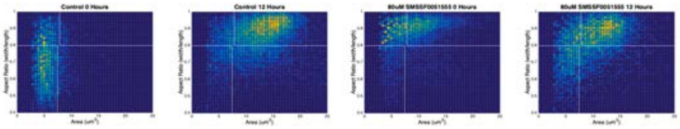	Yes	Yes	Intermediate
4102712	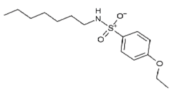	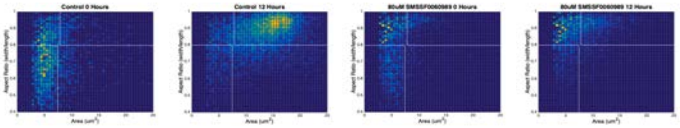	Yes	Yes	Debris
4111118	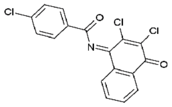	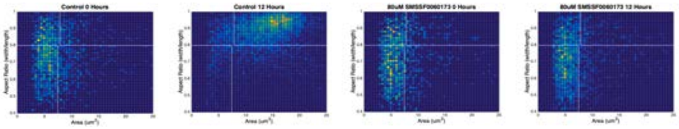	Yes	No	Full
4162926	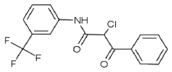	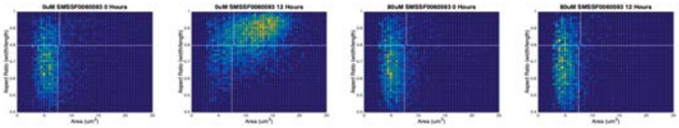	Yes	No	Full

4169124	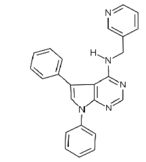	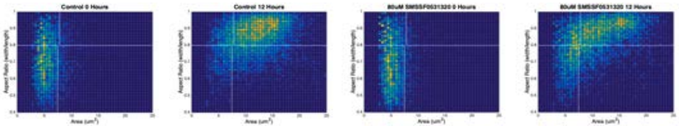	Yes	No	Intermediate
4199189	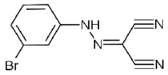	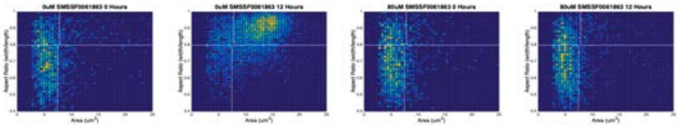	Yes	No	Full
4345313	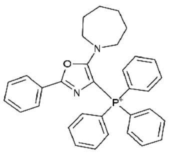	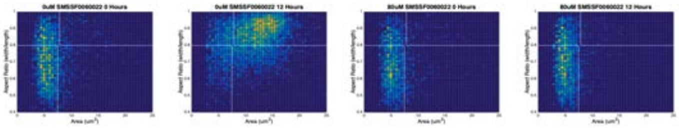	Yes	No	Full
4360162	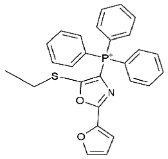	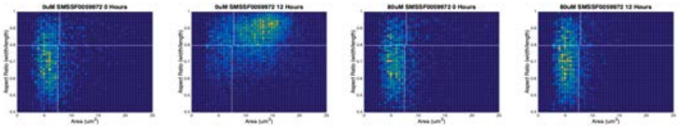	Yes	No	Full
4413055	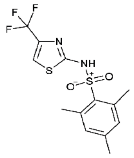	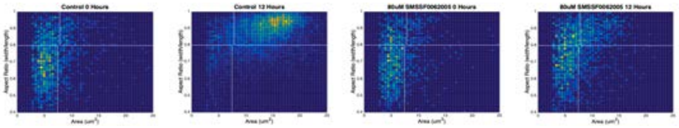	Yes	No	Intermediate
4458495	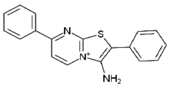	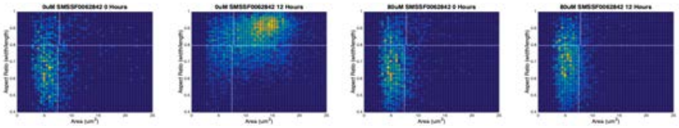	Yes	No	Full
4684724	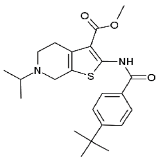	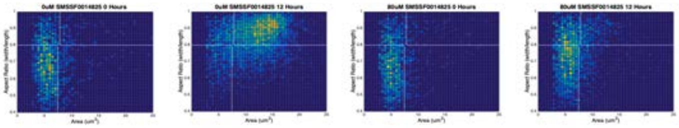	Yes	No	Fulll
4690650	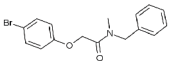	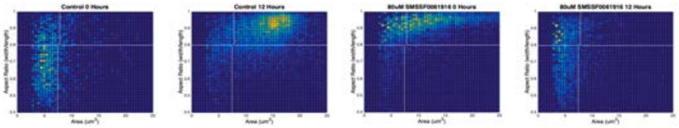	Yes	Yes	Debris
4714160	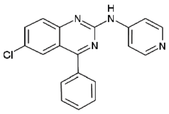	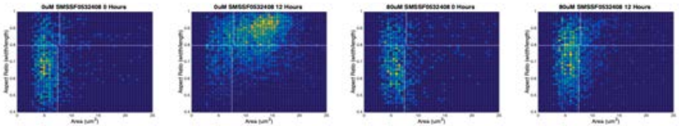	Yes	No	Full
4736829	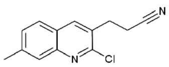	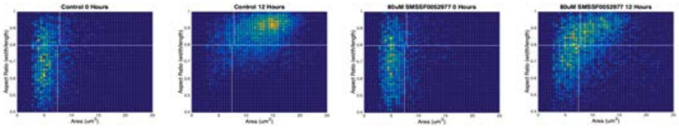	Yes	No	Intermediate
4737365	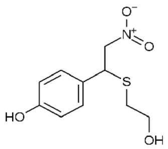	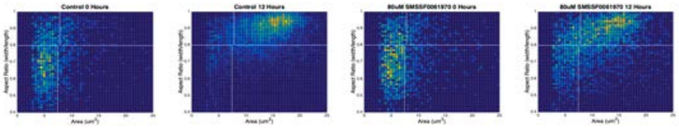	Yes	No	Intermediate

4913014	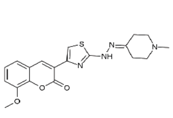	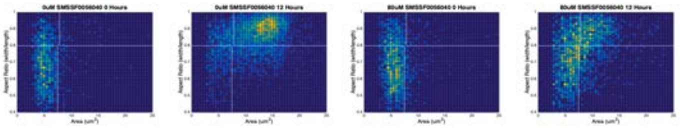	Yes	No	Intermediate
5351098	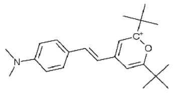	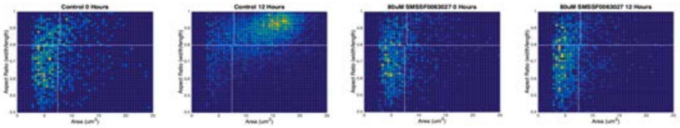	Yes	No	Full
5769808	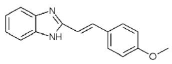	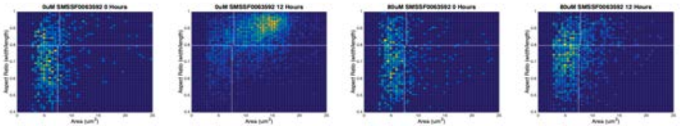	Yes	No	Intermediate
5769972	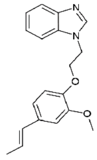	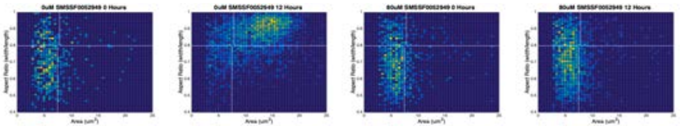	Yes	No	Full
5991625	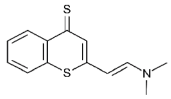	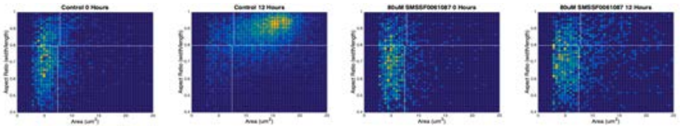	Yes	No	Full
6104220	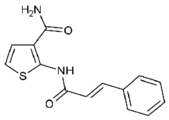	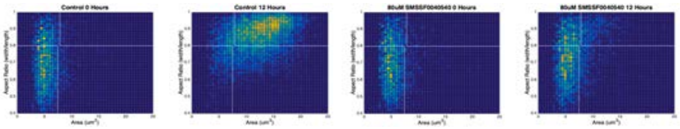	Yes	No	Full
6263817	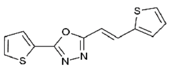	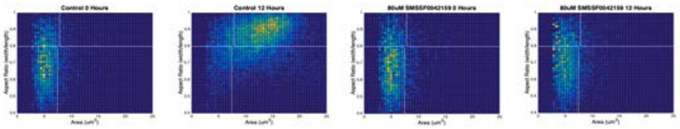	Yes	No	Full
7119035	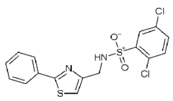	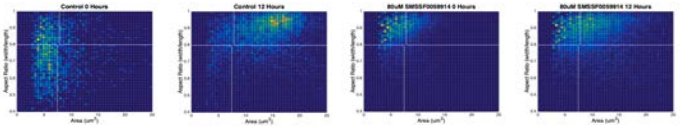	Yes	Yes	Debris
7120750	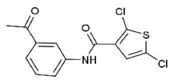	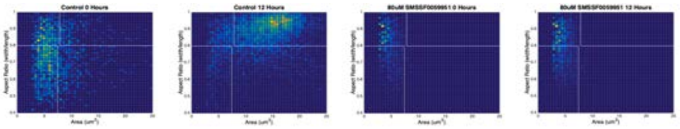	Yes	Yes	Debris
7167022	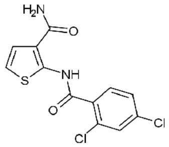	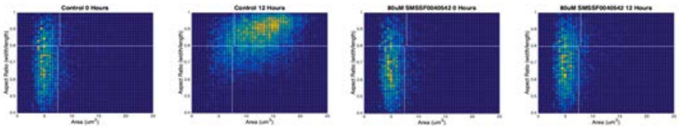	Yes	No	Full
7178481	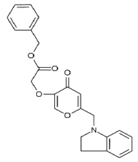	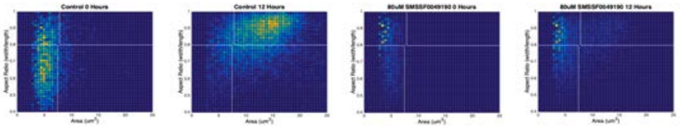	Yes	Yes	Debris

7185492	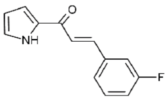	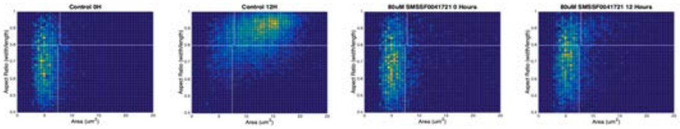	Yes	No	Full
7185494	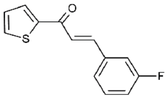	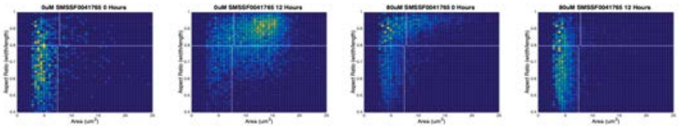	Yes	Yes	Full
7202929	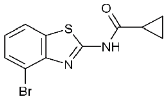	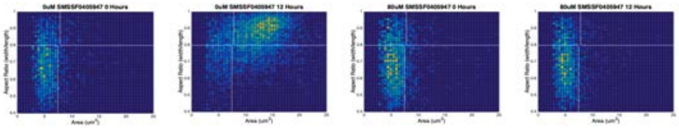	Yes	No	Full
7294823	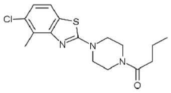	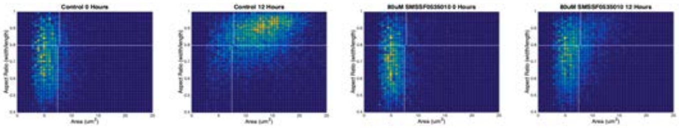	Yes	No	Intermediate
7456205	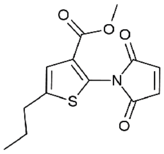	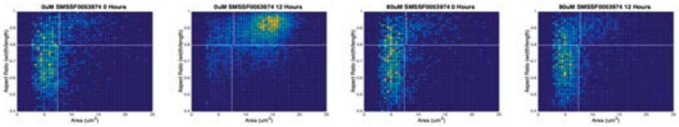	Yes	No	Full
7471335	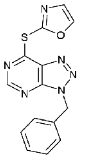	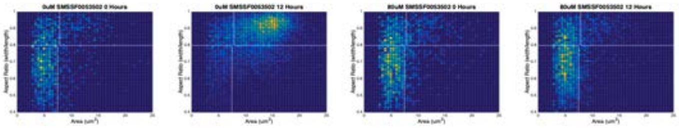	Yes	No	Full
7503016	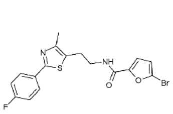	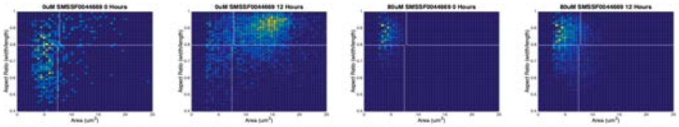	Yes	Yes	Debris
8720355	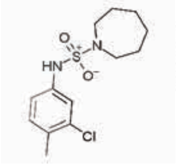	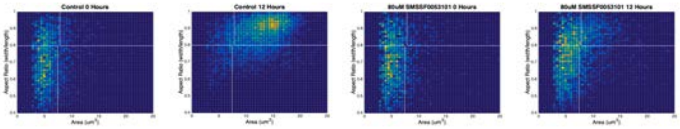	Yes	No	Full
8839503	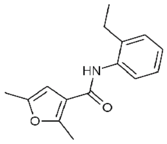	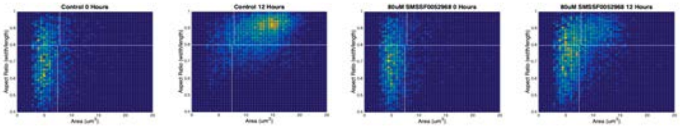	Yes	No	Intermediate
16032794	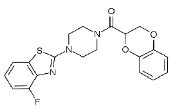	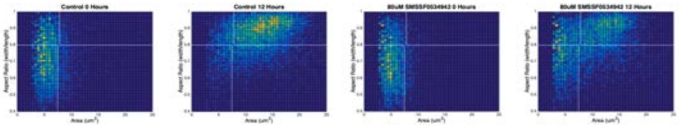	Yes	No	Intermediate
16187117	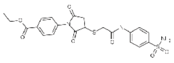	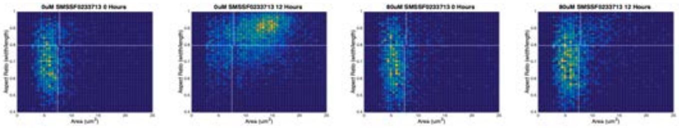	Yes	No	Full

16433415	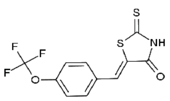	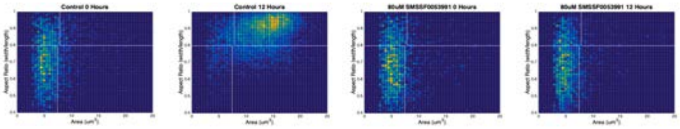	Yes	No	Full
16456321	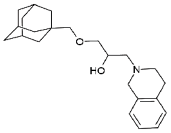	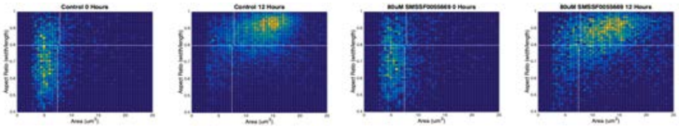	Yes	No	Intermediate
16456368	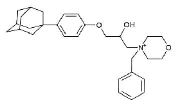	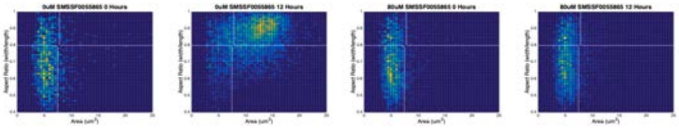	Yes	No	Full
16456942	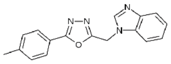	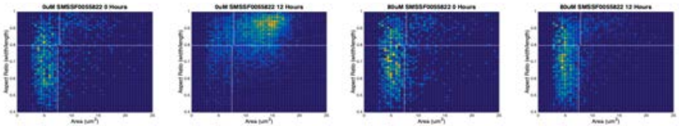	Yes	No	Full
16823609	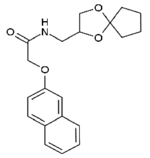	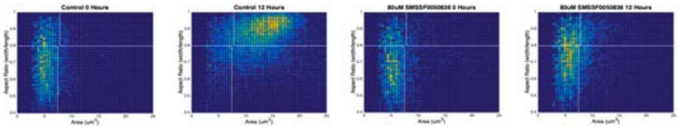	Yes	No	Full
16876071	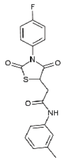	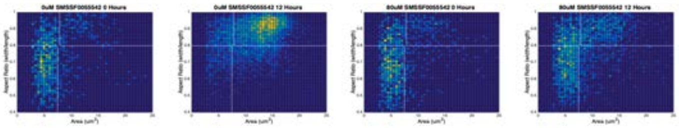	Yes	No	Intermediate
16883840	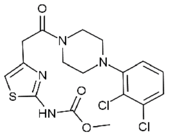	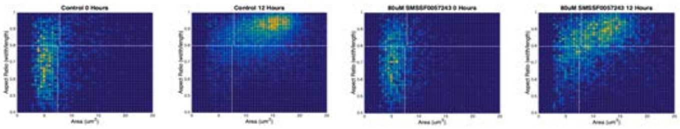	Yes	No	Intermediate
16934160	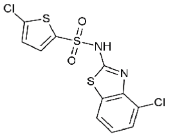	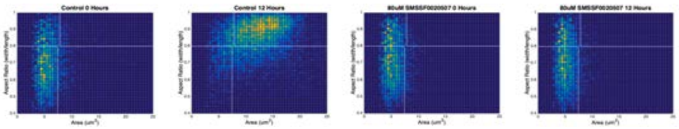	Yes	No	Full
16934170	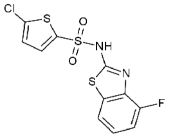	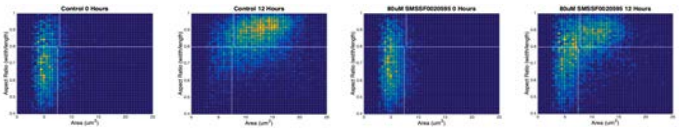	Yes	No	Intermediate
16942479	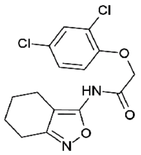	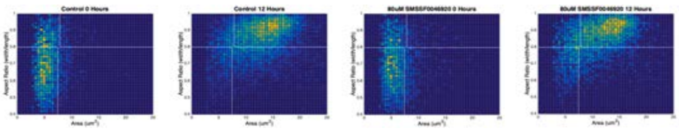	Yes	No	Intermediate
16951979	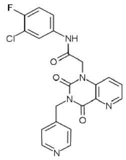	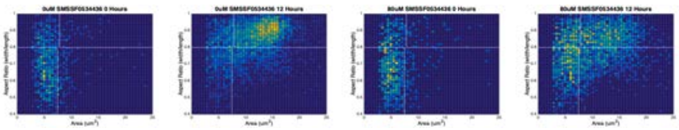	Yes	No	Intermediate

16955786	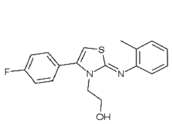	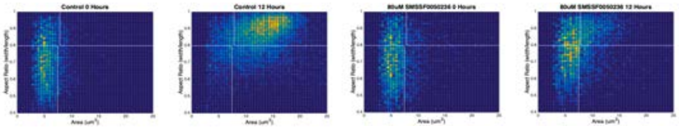	Yes	No	Intermediate
17016619	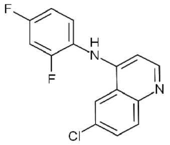	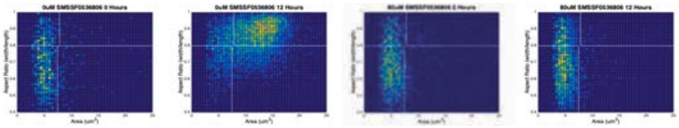	Yes	No	Full
18558862	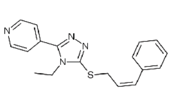	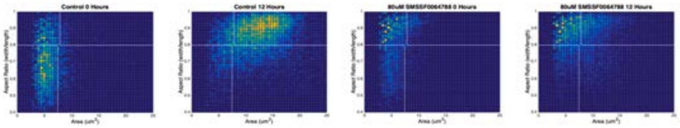	Yes	Yes	Debris
18585549	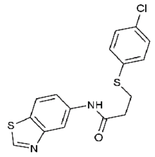	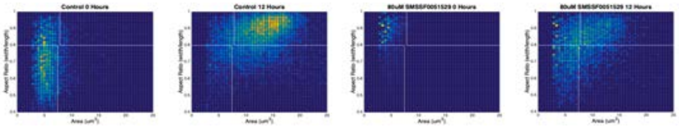	Yes	Yes	Intermediate
18585630	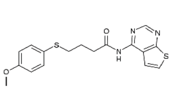	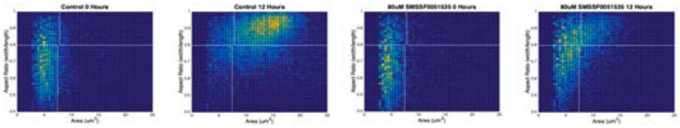	Yes	No	Intermediate
25835862	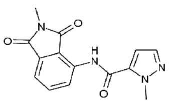	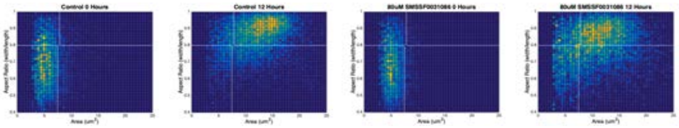	Yes	No	Intermediate
27373469	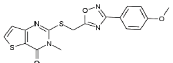	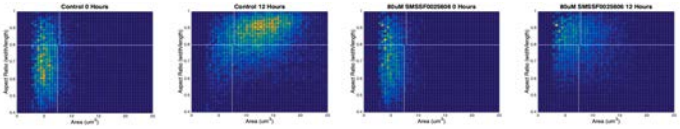	Yes	Yes	Debris
27373907	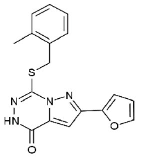	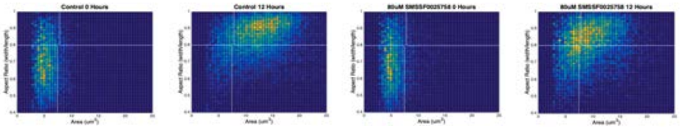	Yes	No	Intermediate
27375613	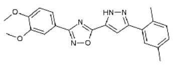	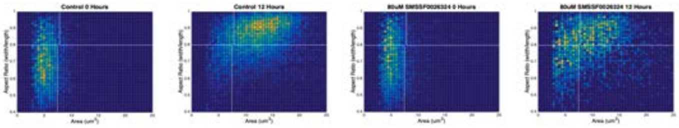	Yes	No	Intermediate
27460748	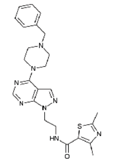	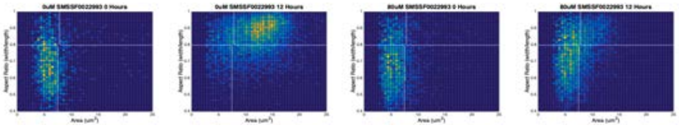	Yes	No	Intermediate
30860168	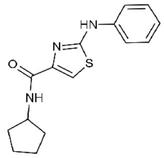	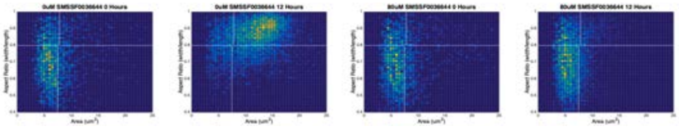	Yes	No	Full

30865811	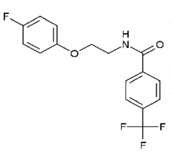	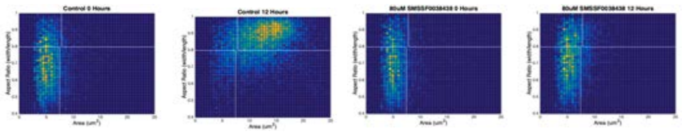	Yes	No	Full
42065409	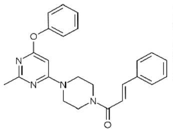	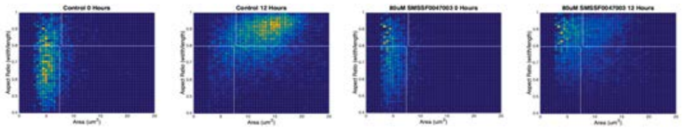	Yes	Yes	Debris
42099839	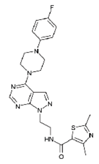	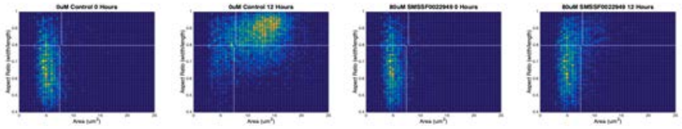	Yes	No	Full
42589452	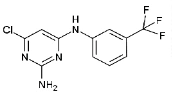	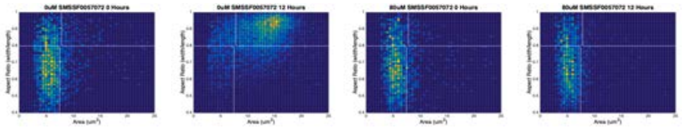	Yes	No	Full
43842355	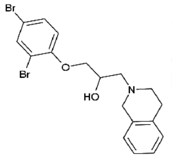	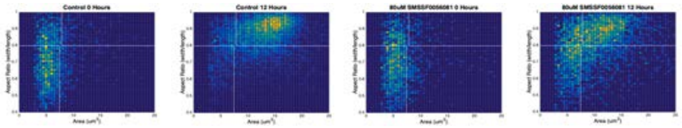	Yes	No	Intermediate
44116743	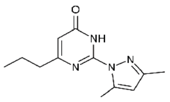	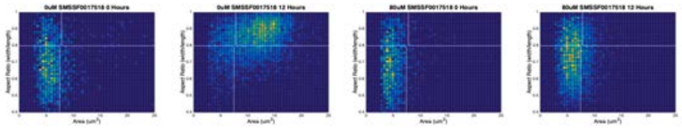	Yes	No	Full
49722107	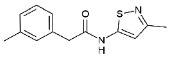	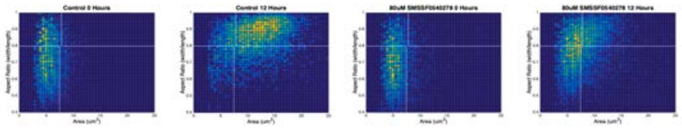	Yes	No	Intermediate
71781394	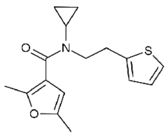	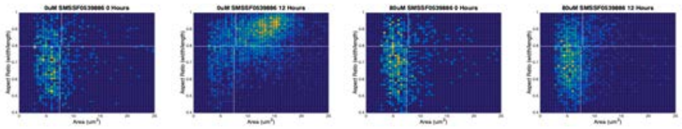	Yes	No	Full
71782913	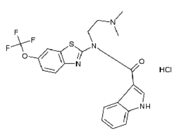	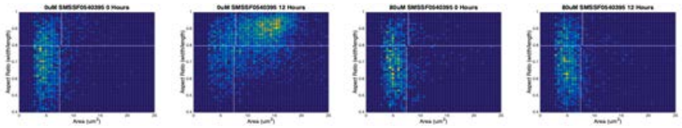	Yes	No	Full
71786870	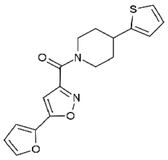	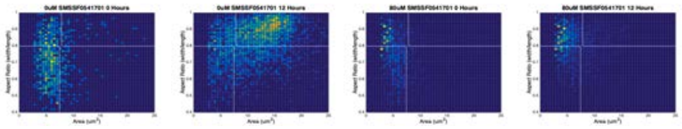	Yes	Yes	Debris
71786876	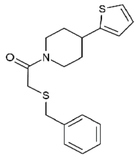	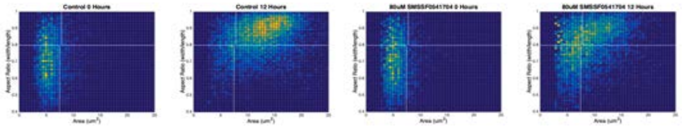	Yes	No	Intermediate

71786877	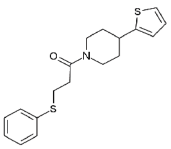	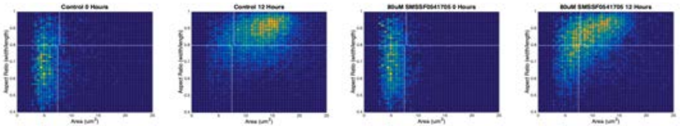	Yes	No	Intermediate
71786985	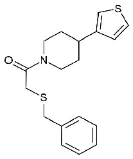	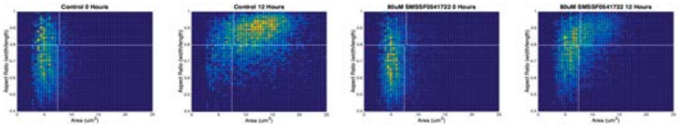	Yes	No	Intermediate
71786990	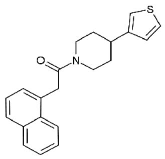	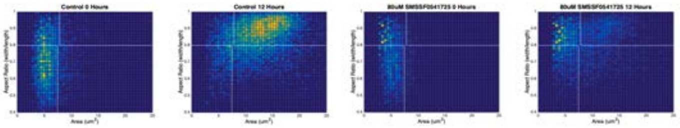	Yes	Yes	Debris
71787916	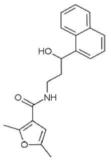	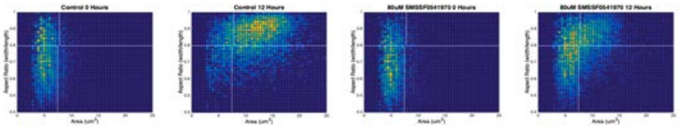	Yes	No	Intermediate
71788301	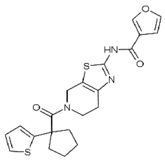	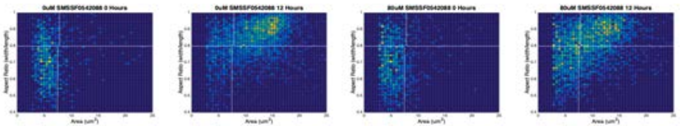	Yes	No	Intermediate
71796286	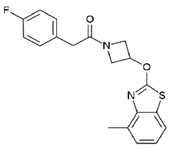	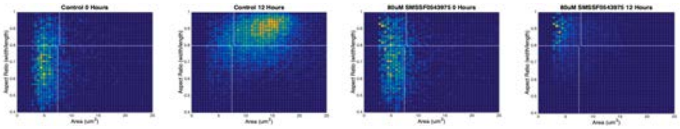	Yes	Yes	Debris
71797038	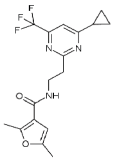	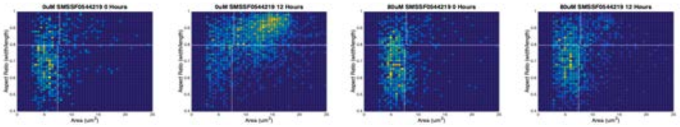	Yes	No	Full
71797126	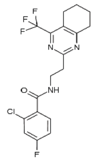	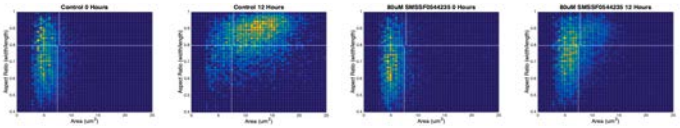	Yes	No	Intermediate
71798856	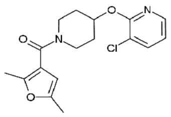	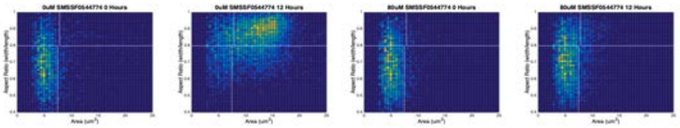	Yes	No	Full
71799766	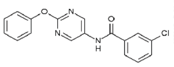	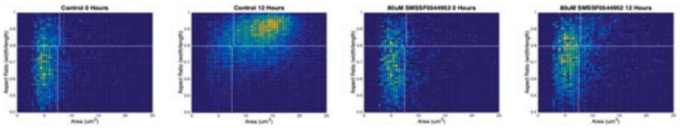	Yes	No	Full
71801965	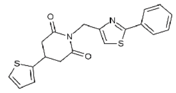	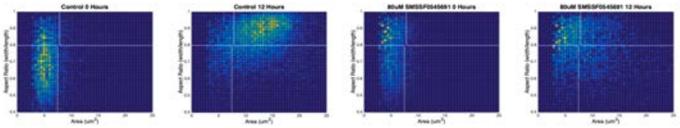	Yes	Yes	Debris

71802096	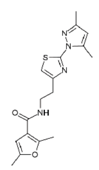	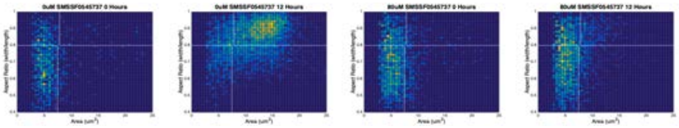	Yes	No	Full
71803023	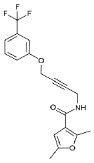	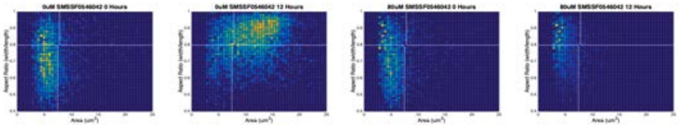	Yes	Yes	Debris
71803055	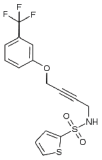	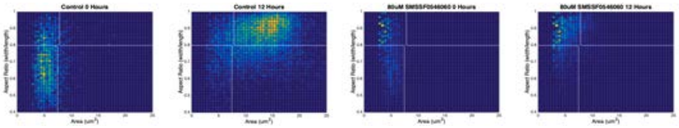	Yes	Yes	Debris
71804107	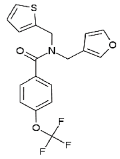	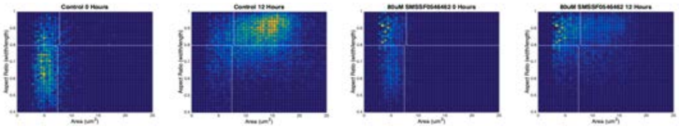	Yes	Yes	Debris
71807060	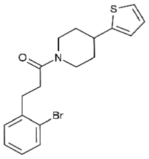	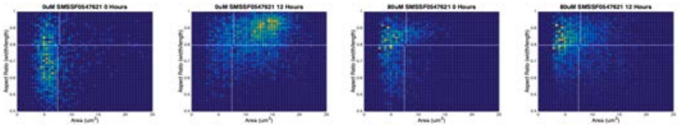	Yes	Yes	Debris
71807075	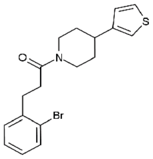	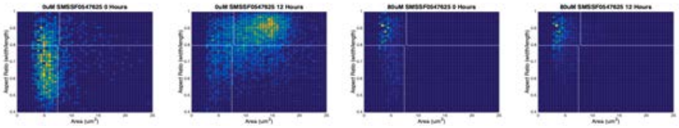	Yes	Yes	Debris
71811049	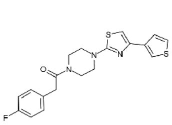	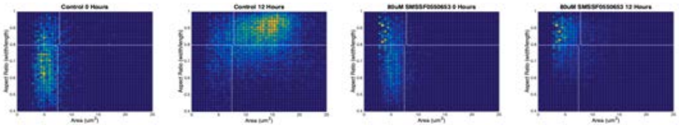	Yes	Yes	Debris
72718563	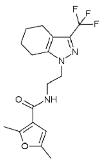	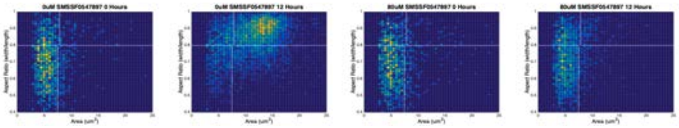	Yes	No	Full
72720194	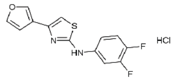	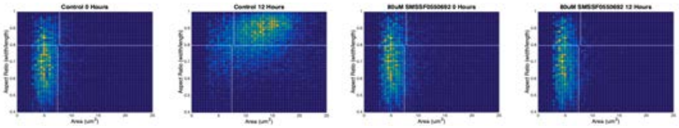	Yes	No	Full
76149097	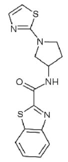	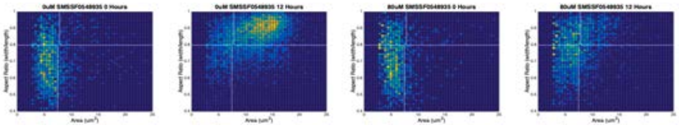	Yes	No	Intermediate
76150259	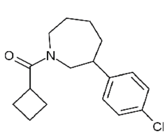	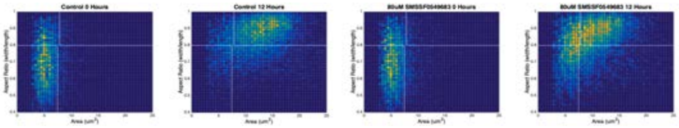	Yes	No	Intermediate

76150266	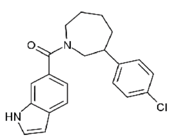	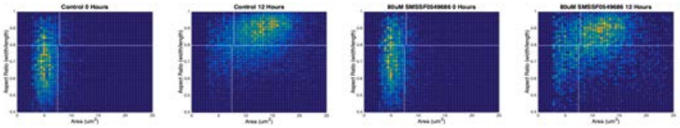	Yes	No	Intermediate
91626508	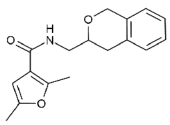	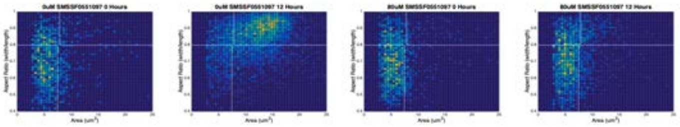	Yes	No	Full
91628556	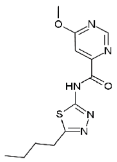	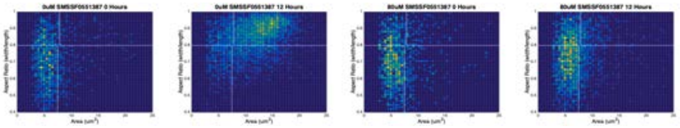	Yes	No	Full
91629266	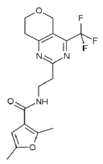	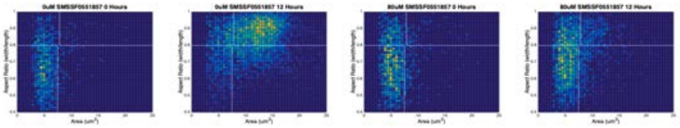	Yes	No	Full
91630328	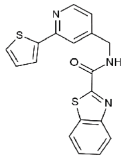	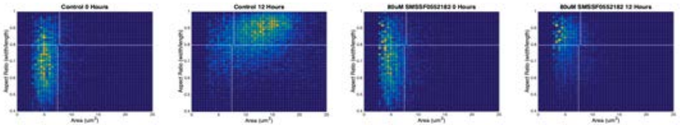	Yes	Yes	Debris
763894	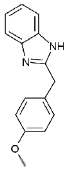	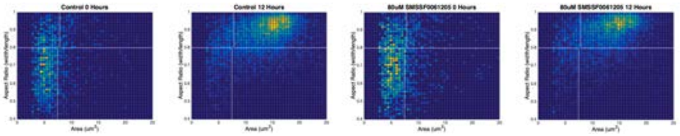	No	No	No
1190482	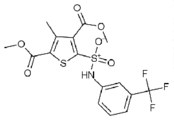	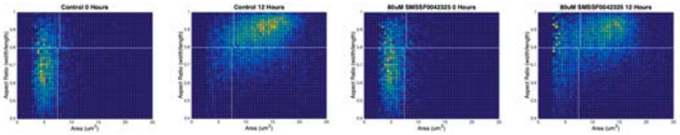	No	Yes	No
1263311	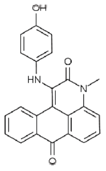	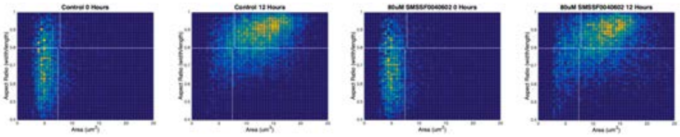	No	No	No
1528700	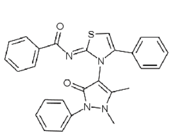	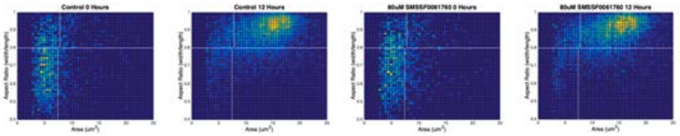	No	No	No
1713451	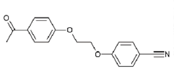	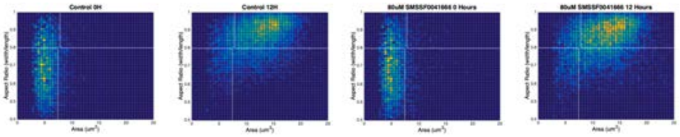	No	No	No
2200363	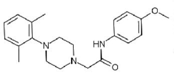	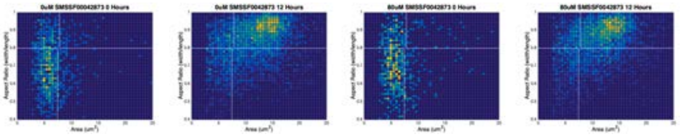	No	No	No

2896856	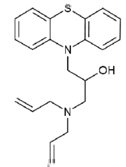	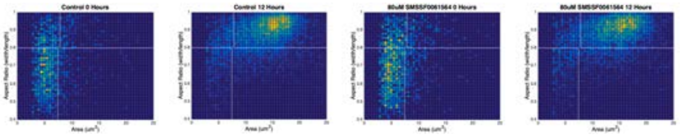	No	No	No
2951953	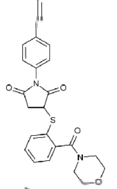	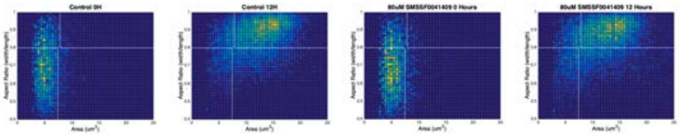	No	No	No
3355288	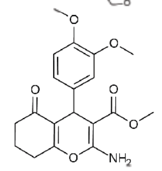	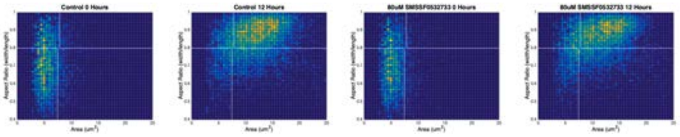	No	No	No
3629287	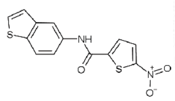	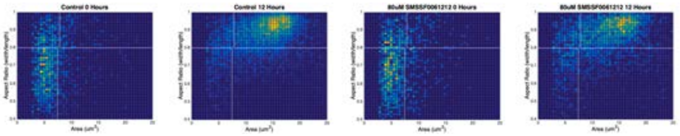	No	No	No
4471873	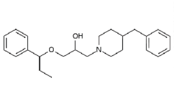	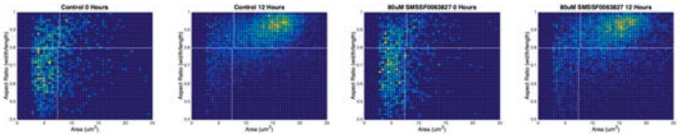	No	No	No
4754109	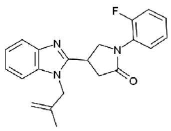	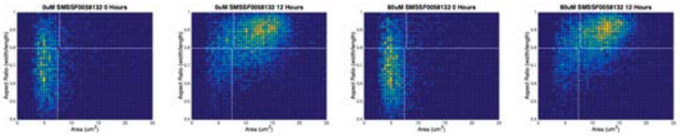	No	No	No
4893324	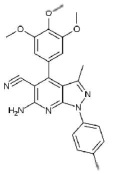	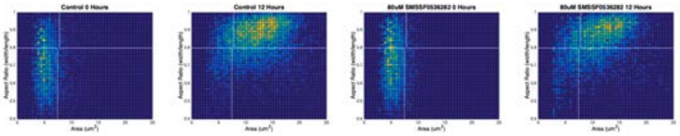	No	No	No
5639066	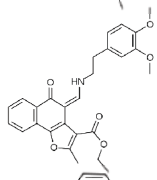	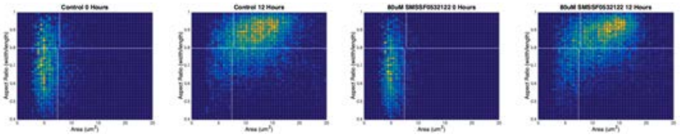	No	No	No
7086348	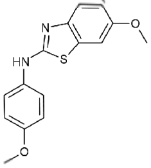	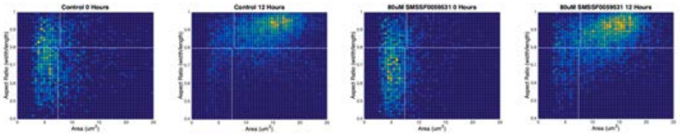	No	No	No
16010290	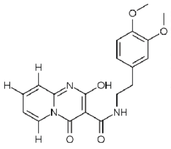	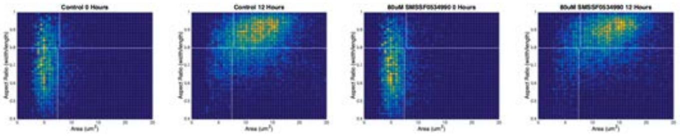	No	No	No
16801811	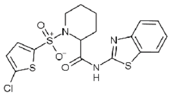	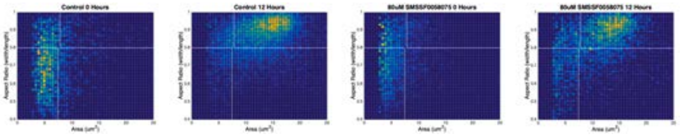	No	Yes	No

16804490	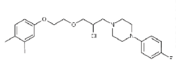	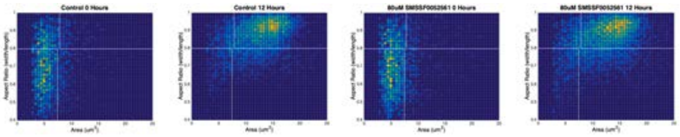	No	No	No
16805904	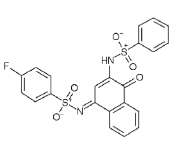	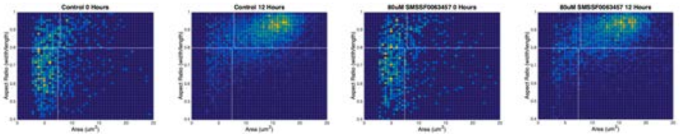	No	No	No
16824016	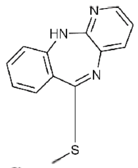	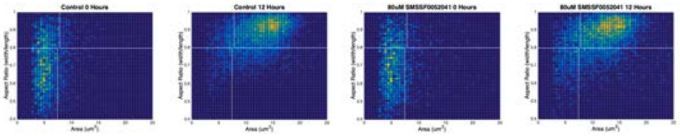	No	No	No
16934153	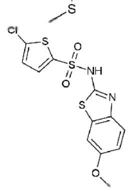	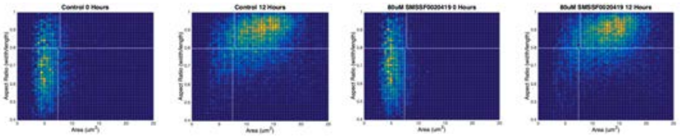	No	No	No
16951905	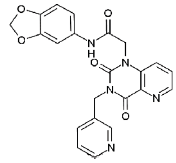	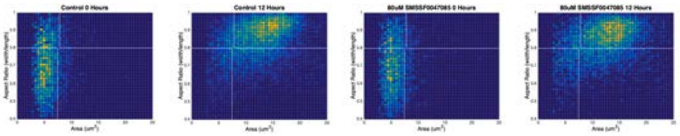	No	No	No
18576765	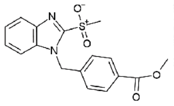	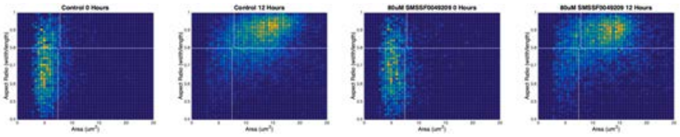	No	No	No
20896311	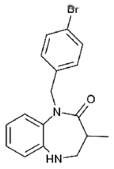	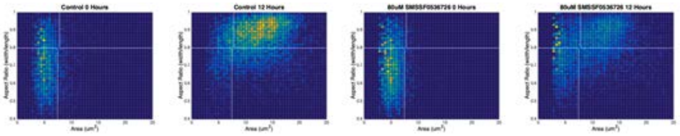	No	Yes	No
24280258	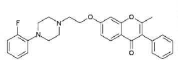	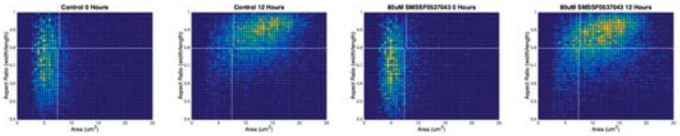	No	No	No
25283140	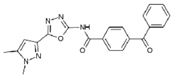	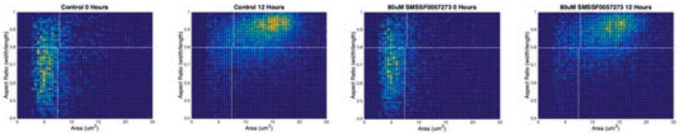	No	No	No
25283686	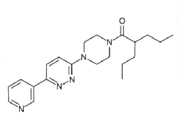	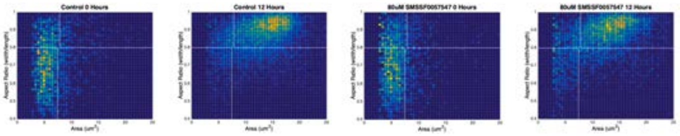	No	No	No
25667819	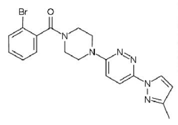	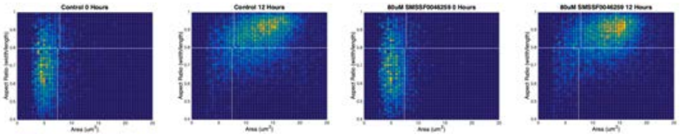	No	No	No

25813521	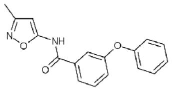	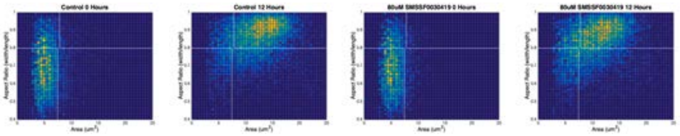	No	No	No
42268033	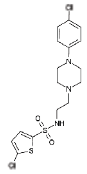	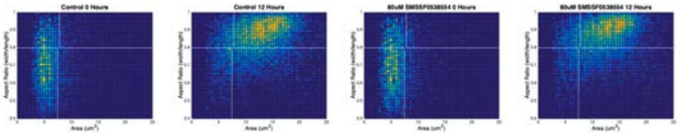	No	No	No
43967671	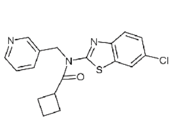	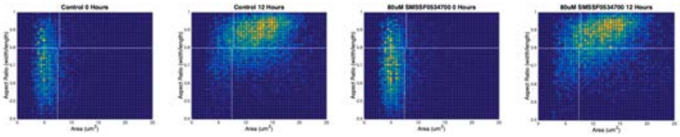	No	No	No
44070699	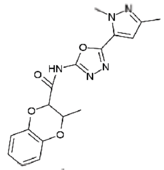	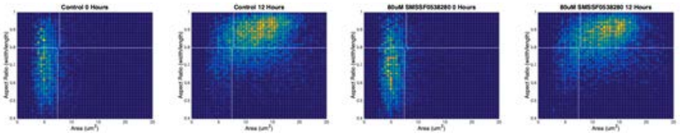	No	No	No
44117349	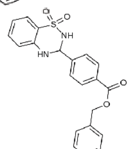	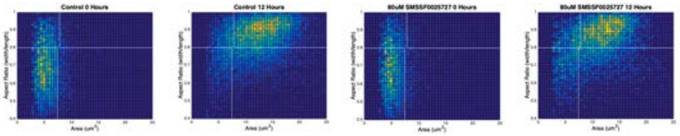	No	No	No
44117394	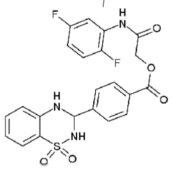	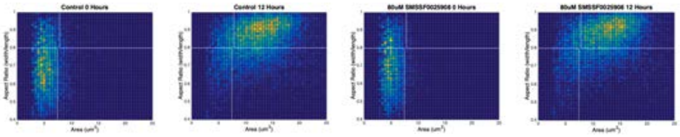	No	No	No
45283772	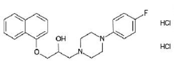	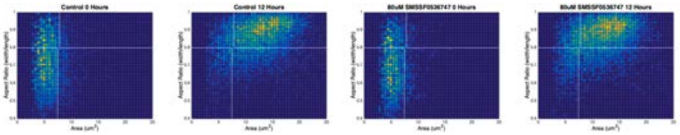	No	No	No
46074589	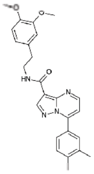	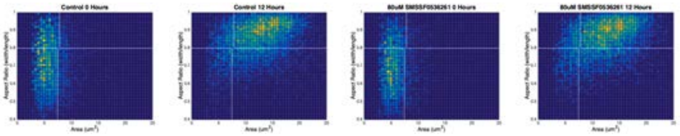	No	No	No
49671361	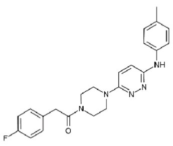	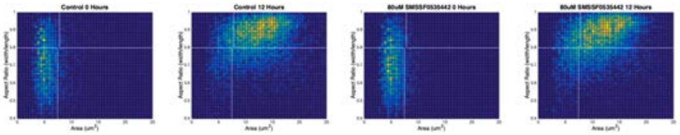	No	No	No
49701731	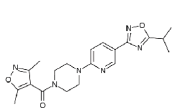	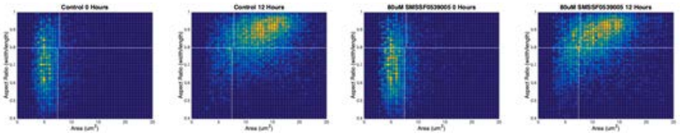	No	No	No
49701828	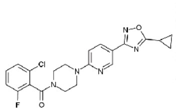	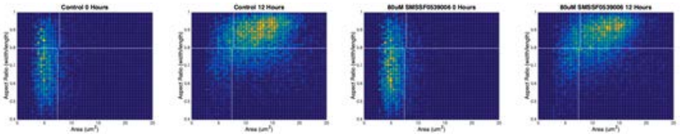	No	No	No

53212169	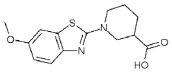	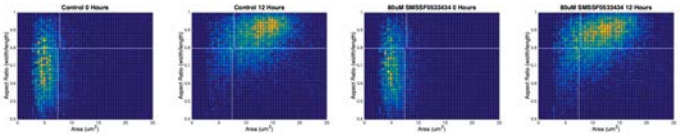	No	No	No
71780502	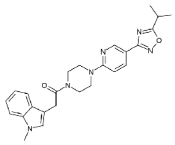	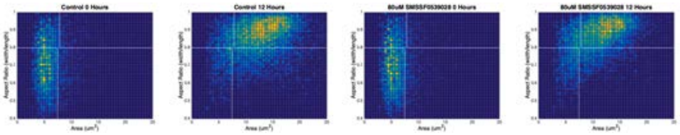	No	No	No
71786989	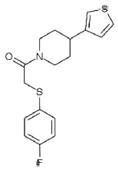	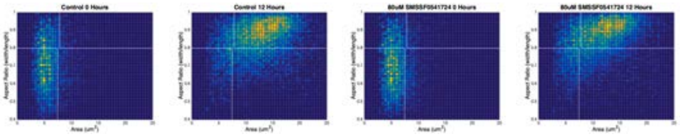	No	No	No
71786992	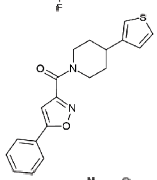	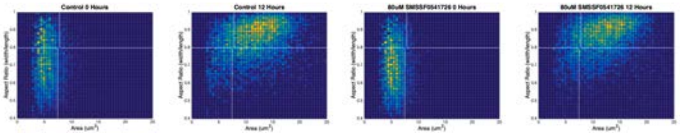	No	No	No
71795763	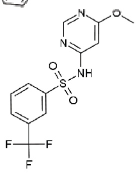	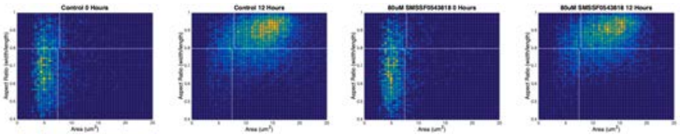	No	No	No
71799471	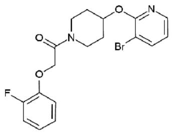	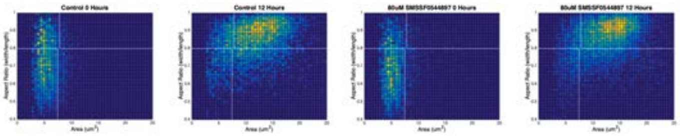	No	No	No
76151751	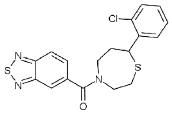	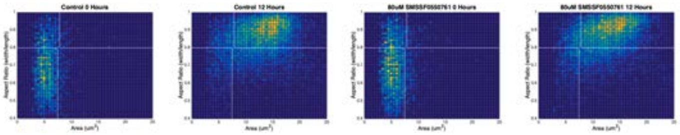	No	No	No
91624614	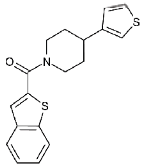	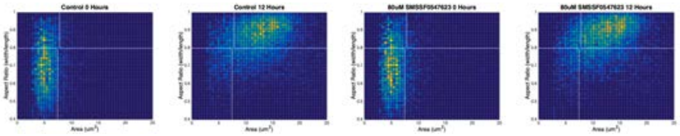	No	No	No
