# Supplementary material for: Discovery of Fungus-Specific Targets and Inhibitors Using Chemical Phenotyping of Pathogenic Spore Germination
Source: mBio. 2021 Jul 27;12(4):e01672-21. doi: 10.1128/mBio.01672-21 (PMC8406298; doi:10.1128/mBio.01672-21)
Supplement: FIG S3 [file mbio.01672-21-sf003.pdf]

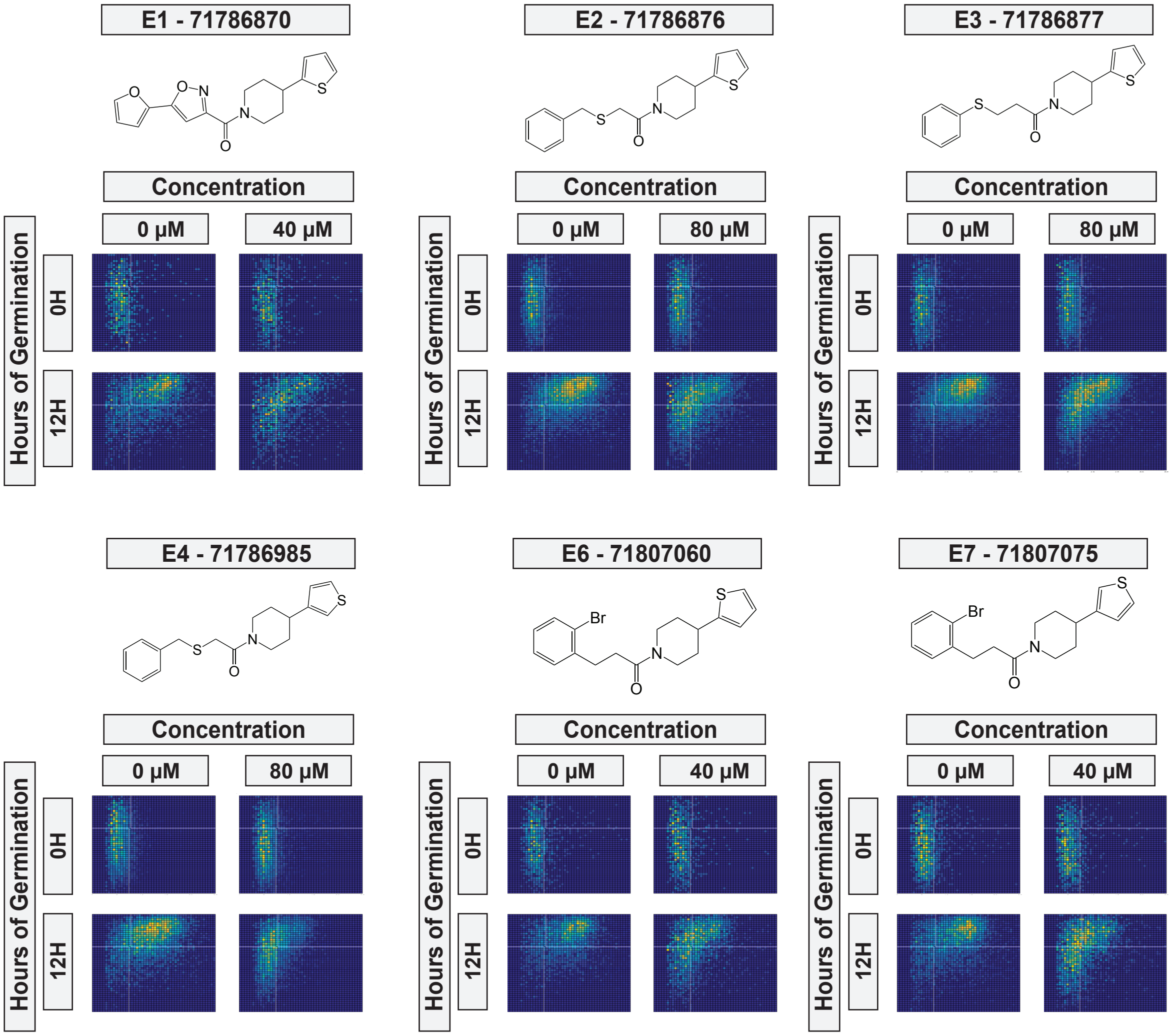

Figure S3. All Group E compounds tested caused an "asynchrony" phenotype. Representative 2-dimensional histograms of ~6,000 spores at phenotypic concentrations of randomly chosen Group E inhibitors.
