# Supplementary material for: Discovery of Fungus-Specific Targets and Inhibitors Using Chemical Phenotyping of Pathogenic Spore Germination
Source: mBio. 2021 Jul 27;12(4):e01672-21. doi: 10.1128/mBio.01672-21 (PMC8406298; doi:10.1128/mBio.01672-21)
Supplement: FIG S4 [file mbio.01672-21-sf004.pdf]

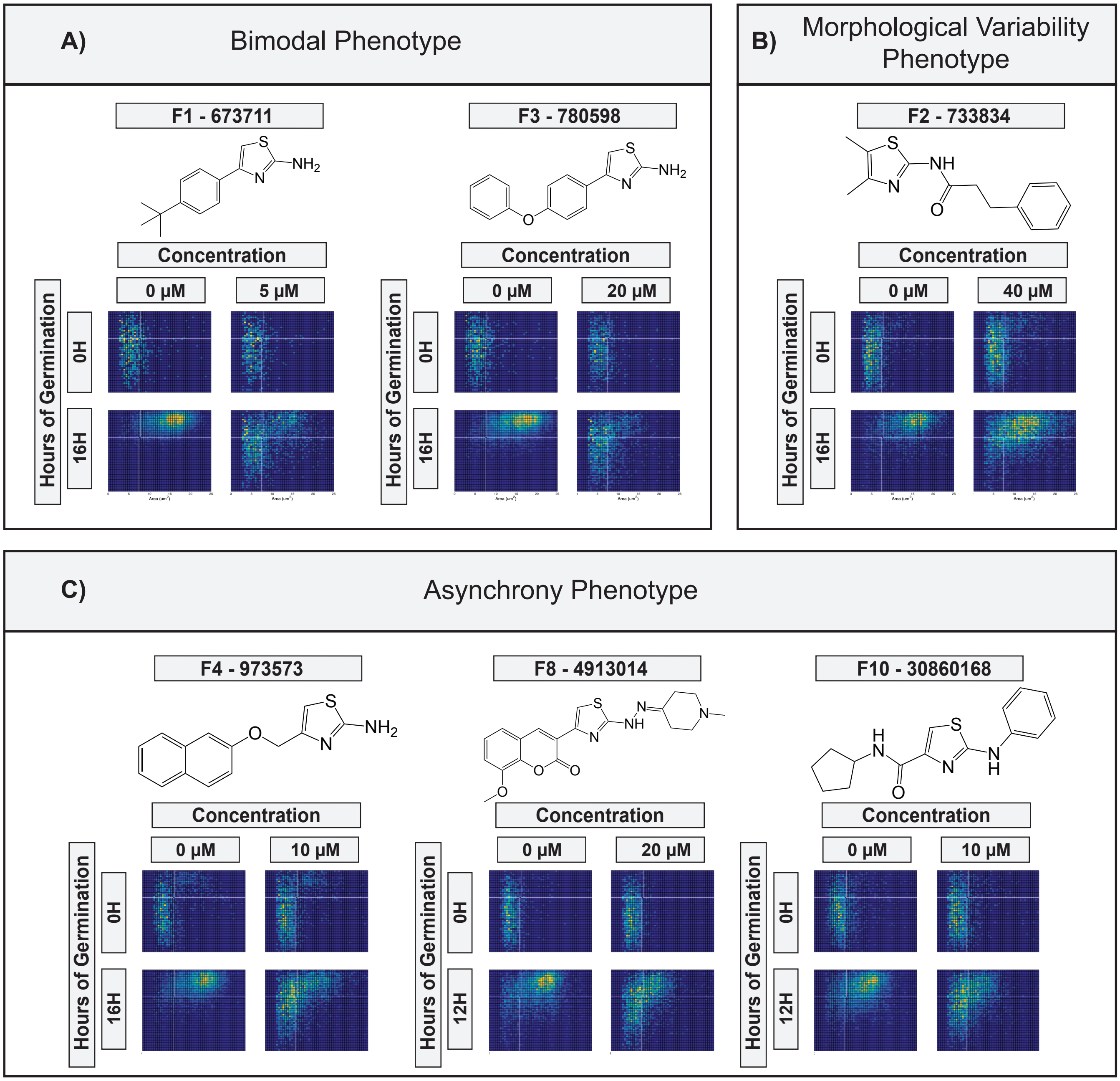

Figure S4. Group F compounds caused three germination inhibition phenotypes. Representative 2-dimensional histograms at phenotypic concentrations of randomly chosen Group F inhibitors. A) F1 and F3 induced a “bimodal” phenotype. B) F2 induced a “morphological variability” phenotype. C) F4, F8, and F10 each induced an “asynchrony” phenotype.
