# Supplementary material for: Discovery of Fungus-Specific Targets and Inhibitors Using Chemical Phenotyping of Pathogenic Spore Germination
Source: mBio. 2021 Jul 27;12(4):e01672-21. doi: 10.1128/mBio.01672-21 (PMC8406298; doi:10.1128/mBio.01672-21)
Supplement: FIG S5 [file mbio.01672-21-sf005.pdf]

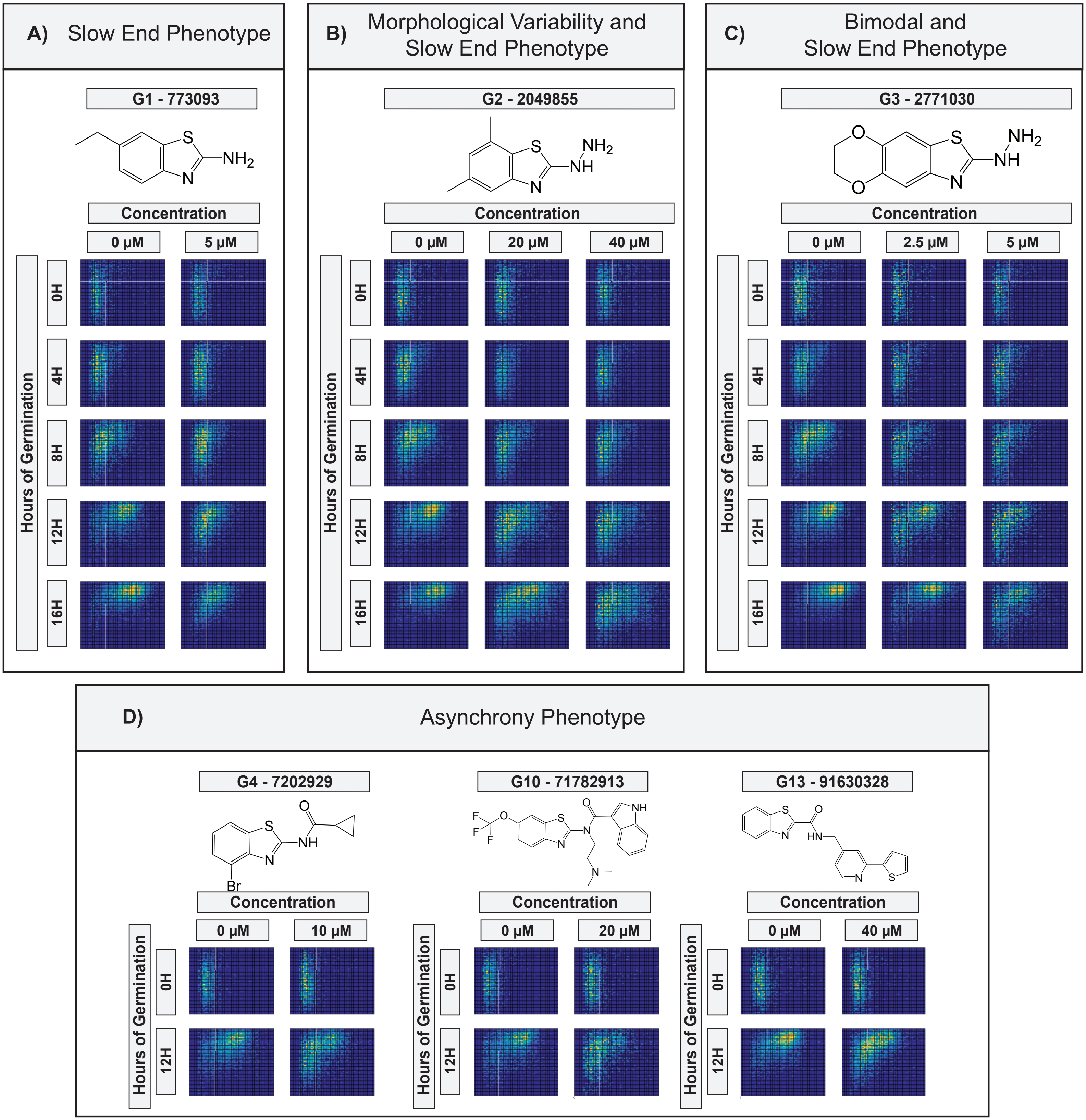

Figure S5. Group G compounds caused 4 germination inhibition phenotypes. Representative 2-dimensional histograms at phenotypic concentrations of randomly chosen Group G inhibitors. A) G1 induced a “slow end” phenotype. B) G2 induced a combination of the “morphological variability” and the “slow end” phenotypes. C) G3 induced a bimodal phenotype (at 2.5 µM) followed by a combination phenotype of “bimodal” and “slow end” at higher concentrations (5 µM). D) G4, G10, and G13 each induced an “asynchrony” phenotype.
